# Visualization of the inflammatory response to injury by neutrophil phenotype categories: Neutrophil phenotypes after trauma

**DOI:** 10.1007/s00068-022-02134-3

**Published:** 2022-11-08

**Authors:** Emma J. de Fraiture, Suus H. Bongers, Bernard N. Jukema, Leo Koenderman, Nienke Vrisekoop, Karlijn J. P. van Wessem, Luke P. H. Leenen, Falco Hietbrink

**Affiliations:** 1 Department of Trauma Surgery, University Medical Center Utrecht, P.O. Box 85500, 3508 GA Utrecht, Netherlands; 2 Department of Trauma Surgery, Sint Antonius Hospital, Utrecht, The Netherlands; 3 Department of Respiratory Medicine, University Medical Center Utrecht, Utrecht, Netherlands; 4 Center for Translational Immunology (CTI), University Medical Center Utrecht, Utrecht, Netherlands

**Keywords:** Trauma, Injury, Neutrophil, Infection, Inflammation

## Abstract

**Purpose:**

The risk of infectious complications after trauma is determined by the amount of injury-related tissue damage and the resulting inflammatory response. Recently, it became possible to measure the neutrophil phenotype in a point-of-care setting. The primary goal of this study was to investigate if immunophenotype categories based on visual recognition of neutrophil subsets are applicable to interpret the inflammatory response to trauma. The secondary goal was to correlate these immunophenotype categories with patient characteristics, injury severity and risk of complications.

**Methods:**

A cohort study was conducted with patients presented at a level 1 trauma center with injuries of any severity, who routinely underwent neutrophil phenotyping. Data generated by automated point-of-care flow cytometry were prospectively gathered. Neutrophil phenotypes categories were defined by visual assessment of two-dimensional CD16/CD62L dot plots. All patients were categorized in one of the immunophenotype categories. Thereafter, the categories were validated by multidimensional analysis of neutrophil populations, using FlowSOM. All clinical parameters and endpoints were extracted from the trauma registry.

**Results:**

The study population consisted of 380 patients. Seven distinct immunophenotype Categories (0–6) were defined, that consisted of different neutrophil populations as validated by FlowSOM. Injury severity scores and risk of infectious complications increased with ascending immunophenotype Categories 3–6. Injury severity was similarly low in Categories 0–2.

**Conclusion:**

The distribution of neutrophil subsets that were described in phenotype categories is easily recognizable for clinicians at the bedside. Even more, multidimensional analysis demonstrated these categories to be distinct subsets of neutrophils. Identification of trauma patients at risk for infectious complications by monitoring the immunophenotype category is a further improvement of personalized and point-of-care decision-making in trauma care.

**Supplementary Information:**

The online version contains supplementary material available at 10.1007/s00068-022-02134-3.

## Background

Patients with severe injuries are at risk for (severe) infectious complications. Due to advances in the organization of trauma care, hemorrhage control, surgical approaches and resuscitation, overall mortality after trauma has declined up to 75% in recent decades [1–3]. With increased early survival rates, up to 30–50% of multitrauma patients develop an infectious complication, which creates new challenges in terms of diagnostics and treatment in longitudinal management of trauma patients [4]. Infections are now the most common complications affecting trauma patients and are an increasing and substantial cause of morbidity, contributing to a mortality rate of 5–8% after trauma [1, 5, 6]. Prior research has shown that the risk of infections after trauma is determined by injury-related tissue damage leading to an inflammatory response initiated by damage-associated molecular patterns (DAMPs) [7, 8]. The innate immune system, in particular the neutrophil compartment, is essential in tissue repair and in the first line of defense against invading pathogens. Therefore, these cells are very sensitive for activation by DAMPs [9–11]. An imbalance in the neutrophil compartment after trauma and a subsequent dysregulation of the immune response predisposes patients to infections [12].

Recently, it became possible to measure the neutrophil phenotype at the patient’s bedside in the acute setting with an automatic point-of-care flow cytometry approach [13]. In our center, the phenotypical and functional analysis of the neutrophil compartment has been integrated into clinical practice, allowing early immune monitoring to aid diagnosis and prognosis of aberrant innate immune responses. This could help in treatment choices in this very heterogenous patient population.

During acute inflammation, neutrophils can be divided into different subsets, based on the expression of specific surface proteins (CD16/FcγRIII and CD62L/L-selectin) [14]. A correlation was shown between injury severity and the percentage of circulating CD16^dim^ neutrophils [15]. In addition, we have previously shown that a decreased responsiveness of neutrophils to formyl-peptides (fMLF) immediately after trauma was related to the development of septic shock 5–8 days later [16]. Both parameters provide information on the inflammatory response to injury.

The primary objective of this study was to define immunophenotype categories, based on visual recognition of neutrophil subsets by CD16/CD62L expression, and to investigate by algorithm based analysis if these categories are valuable to interpret the inflammatory response to trauma. The secondary goal was to determine if the immunophenotype categories correlate with potentially pre-existing patient factors, the severity of injuries, and clinical outcome of trauma patients, in terms of infectious complications, duration of hospital admission and mortality.

## Methods

### Study design

A cohort study was conducted in patients presented at the trauma bay of the UMC Utrecht between January 1, 2020 and March 1, 2021. The included patients routinely underwent neutrophil phenotyping as a standard of care procedure during resuscitation. Flow cytometry data were prospectively gathered, whereas patient data were retrospectively collected from the electronic patient files and the trauma registry (a prospectively collected database) and combined with the flow cytometry data. The STROBE guideline was used to ensure proper reporting of methods, results, and discussion (Online Resource 1). This study was approved by the University Medical Centre Utrecht ethical review committee (21-224_AQUIretro). The processing and storage of data were in accordance with privacy and ethics regulations.

## Patients

Included patients were trauma patients of 18 years and older, presented at the trauma bay in the emergency department of the University Medical Center Utrecht (UMCU). The criteria for inclusion were trauma patients with injuries of any severity who underwent diagnostic blood sampling as part of the standard-of-care diagnostic workup during acute trauma care. No exclusion criteria based on patient characteristics nor injury severity were formulated, because the focus of this project was to analyze the immune response as early as possible after trauma, and to investigate if patient and/or injury characteristics affect this inflammatory response. Patients who were discharged from the hospital directly after presentation were identified as a non-significantly injured reference group. Patients transferred from other hospitals to the UMC Utrecht > 24 h after trauma were excluded because the first inflammatory response would not reflect a measurement < 24 h after injury. Patients without interpretable results of measurement on the flow cytometer were also excluded. Examples of uninterpretable results are a too low number of analyzed cells, a malfunction or technical error of the machine (e.g., a clog or bubble in the system, or failed lysis of red blood cells), or missing activation data. Immunocompromised patients were not excluded, because the possible impact of immunosuppressive medication on the inflammatory reaction to trauma has not been investigated earlier.

### Healthy controls

Blood from healthy controls was obtained via the “mini donor service” at the UMC Utrecht. Healthy controls were between 18–65 years old and were from both sexes. The protocol for blood withdrawal was approved by the Medical Ethical Committee of the UMCU (study approval number 18/774) and healthy controls gave informed consent. Healthy controls were not compared to included patients, but were used to define the CD16/CD62L expression pattern in healthy controls.

### Study procedures

All included trauma patients underwent blood sampling and subsequent analysis by an automated flow cytometer directly after presentation at the trauma bay. Time of arrival since trauma was not registered. Previous studies in this region showed that patients arrive at the hospital between 20 and 60 min after trauma [17, 18]. Blood is drawn within 10 min after presentation at the trauma bay and directly placed in the flow cytometer. Analysis by the flow cytometer takes 18 min, so the receptor expression as measured with the flow cytometer reflects the first immune response in the blood.

#### Flow cytometer procedure

A blood sample in a 4 mL sodium-heparin tube (Greiner Bio-One GmbH, Kremsmünster, Austria) was drawn as part of the standard-of-care diagnostic work-up during acute trauma care. Neutrophil markers were measured (e.g., CD16 and CD62L) by means of a direct and automated flow cytometric measurement. After venipuncture the blood tube was directly placed into the AQUIOS CL^®^ “load & go” flow cytometer (Beckman Coulter Life Sciences, Miami, FL, USA) by the staff of the trauma team. This flow cytometer is programmed to run an automatic work-up and analysis protocol. First, the machine pipettes the blood into a 96-deep well plate. The blood is then stained for 15 min with 18 µL customized antibody mix for neutrophils [13, 19]. The antibody mix used was the same as described by Spijkerman et al. [15], i.e. CD16-FITC (clone 3G8), CD11b-PE (clone Bear1), CD62L-ECD (clone DREG56), CD10-PC5 (clone ALB1) and CD64-PC7 (clone 22; all clones from Beckman Coulter). Tests were executed in the presence and absence of the formyl peptide *N*-formyl-norleucyl-leucyl-phenylalanine (fNLF) that is used to activate neutrophils by interaction with its formyl peptide receptor (BioCat GmbH, Heidelberg, Germany) at an end concentration of 10^−5^ M. It is important to emphasize that fNLF was used, because in contrast to fMLF this formyl-peptide is not sensitive to inactivation by oxidation and is equally potent in activating neutrophils [13]. The lysis is stopped after 30 s by adding 100 µL AQUIOS Lysing Reagent B, followed by aspiration and analysis through the flow cell.

Data generated by the flow cytometer were stored as a summary.pdf file and a flow cytometry standard (.lmd) file, linked by date of analysis and patient number on a secured drive of the hospital. On the summary.pdf file flow cytometry gating plots, percentages of CD16^dim^ and CD62L^dim^ neutrophils, and numeric expression of neutrophil surface markers were displayed. Before exporting the summary.pdf file, the granulocyte gate based, on forward scatter-sideward scatter, was manually checked and adjusted on the (software of the) flow cytometer if necessary. Eosinophils were excluded based on low CD16 expression. TheCD16/CD62L quadrant gates were fixed and thus identical for each sample. The original flow data were exported in.lmd files. The.lmd files were exported and analyzed using Cytobank Premium (www.cytobank.org, a web-based flow cytometry analysis platform; Beckman Coulter, Indianapolis, IN, USA).

### Neutrophil subset categories

By visual assessment of all two-dimensional CD16 (*x*-axis)/CD62L (*y*-axis) dot plots immunophenotype categories were defined by visual pattern recognition, without the use of preset gates. The choice for 2D analysis was made because refitting cut-off values for dim/low expression into a single parameter for clinical use proved to be challenging and time-intensive for clinicians and visual pattern recognition was effectively easier and faster for surgeons to use in de clinical setting. Moreover, interpersonal variation in gating for studying the expression of single general markers and quality of measurement of antibody staining could lead to artificial differences in MFI results, while the visual expression patterns remain unchanged. The neutrophil subset categories were defined as follows. First, neutrophil population phenotypes were described by one researcher and ranked in ascending order on the basis of increased deviation from healthy receptor expression (by criteria as described in the Results section), solely based on neutrophil CD16 and CD62L expression. The expression of these markers was not related to cell size, granularity within the neutrophil population or responsiveness to fNLF. Two independent researchers repeated the distribution of the dot plots into the defined phenotype categories. In case of disagreements, a discussion (blinded for the outcome) leaded to consensus about all dot plots. All.pdf files were manually assessed and categorized into one of the immunophenotype categories by at least two independent researchers.

### Cluster analysis by FlowSOM

To validate the earlier mentioned two-dimensional approach to immunophenotyping neutrophil categories, an automated clustering (FlowSOM) analysis was performed. FlowSOM is a high-dimensional clustering and visualization algorithm, based on a self-organizing map. Neutrophils were identified as follows: (1) Granulocytes were gated based on forward-/sideward-scatter, identical to the gating strategy on the AQUIOS. (2) The granulocytes were analyzed through FlowSOM by using 6 metaclusters and 64 clusters. (3) The neutrophil metacluster was identified by CD16/CD11b expression. (4) To identify neutrophil subsets the neutrophil metacluster from all inactivated samples (without fNLF) was analyzed through FlowSOM again, this time using a previously build FlowSOM, based on a dataset of FACS samples from five volunteers before and during acute inflammation (experimental endotoxemia or LPS-challenge), using 3 metaclusters and 16 clusters. Blood analyzed before LPS-challenge is representative for homeostatic neutrophil subsets. During a LPS challenge in healthy individuals banded (CD16^dim^/CD62L^bright^) and hypersegmented (CD16^bright^/CD62L^dim^) subsets appear in the blood [20]. These subsets are a robust representative for the subsets that occur in severely injured trauma patients.

The FlowSOM algorithm based on LPS-challenge data revealed three neutrophil metaclusters in trauma patients. Metacluster 1 was identified as mature neutrophils (CD16^bright^/CD62L^bright^), metacluster 2 was identified as banded neutrophils (CD16^dim^/CD62L^bright^) and metacluster 3 was identified as hyper-segmented neutrophils (CD16^bright^/CD62L^dim^). The percentages of cells in each MC were exported (example of FlowSOM result overview in Online Resource 2). For all mentioned FlowSOM analyses, all markers of the flow cytometry panel were used (CD10, CD11b, CD16, CD62L, CD64).

To investigate if the defined immunophenotype categories represented significantly different neutrophil populations, the percentages of neutrophil populations in MC 1, 2 and 3 and the responsiveness to fNLF of the neutrophil population as a whole, displayed as the ratio fNLF+/fNLF− for each receptor were compared between the predefined immunophenotype categories.

### Other parameters and endpoints

All other clinical parameters and endpoints were retrospectively extracted from the trauma registry and completed with data from the hospital administration database. Age, sex, and comorbidities were assessed as possible pre-existing patient factors affecting the immunophenotype after trauma. Comorbidities were classified in the ASA Physical Status Classification System [21]. Infectious complications were defined as the report of an infectious complication in the patient file in combination with antibiotic treatment according to the hospital guidelines.

### Statistical analysis

Data were collected in a database and were analyzed with Stata 14 (StataCorp. 2015. *Stata Statistical Software: Release 14.* College Station, TX: StataCorp LP). Variables were analyzed using Kruskal–Wallis *H* test. Post hoc testing was performed using Dunn’s multiple comparison test with Bonferroni correction. A *p* value of < 0.05 was used for statistical significance.

## Results

**Fig. 1:**
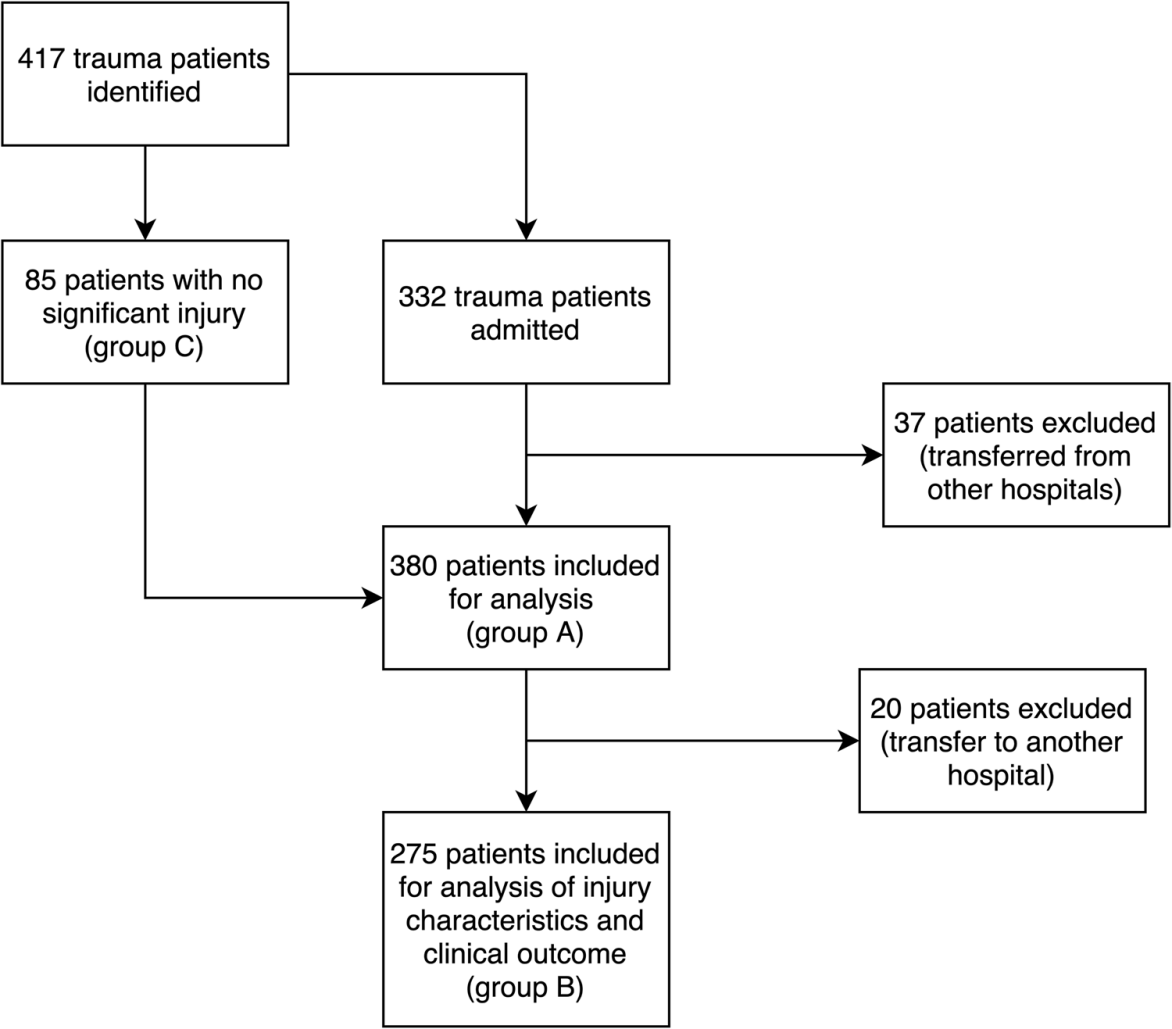
Flowchart of patient inclusion and data processing

In total, 417 trauma patients were identified based on trauma registration and measurement on the flow cytometer, of which 380 were included for analysis. After inclusion, one measurement had to be excluded due to an incorrect display of values. For 20 patients, follow-up was not possible, as these patients were transferred to another hospital after admittance and acute care in the UMCU trauma bay. As shown in Fig. 1, three different groups of patients were defined: all trauma patients (group A), who were then divided into: the significantly injured patients, as defined by their admittance to the hospital, (group B) and the patients without significant injury who were not admitted (group C). An overview of patient characteristics is described in Table 1.

**Table 1:** Overview of patient and injury characteristics for all trauma patients (group A), all significantly injured patients (group B) and all insignificantly injured patients (group C)

	All patients (group A) *N*	Significant injury (group B) *N* (%)	No significant injury (group C) *N* (%)
Total	380	295	85
Sex			
Male	264	202 (68)	62 (73)
Female	116	93 (32)	23 (27)
Age			
Mean ± SD	51.8 ± 20.70	53.9 ± 20.85	44.6 ± 18.54
Median (range) IQR	53 (18–99) 33–70	55 (18–99) 35–71	41 (18–90) 29–60
ASA-classification			
1	145	98 (34)	47 (55)
2	153	125 (43)	28 (33)
3	75	65 (22)	10 (12)
4	4	4 (1)	
Immunocompromised	9	6 (67)	3 (33)
ISS			
Mean ± SD		14.6 ± 11.14	NA
Median (range)		13 (1–75)	
IQR		8–19	

### Immunophenotype categories

**Fig. 2:**
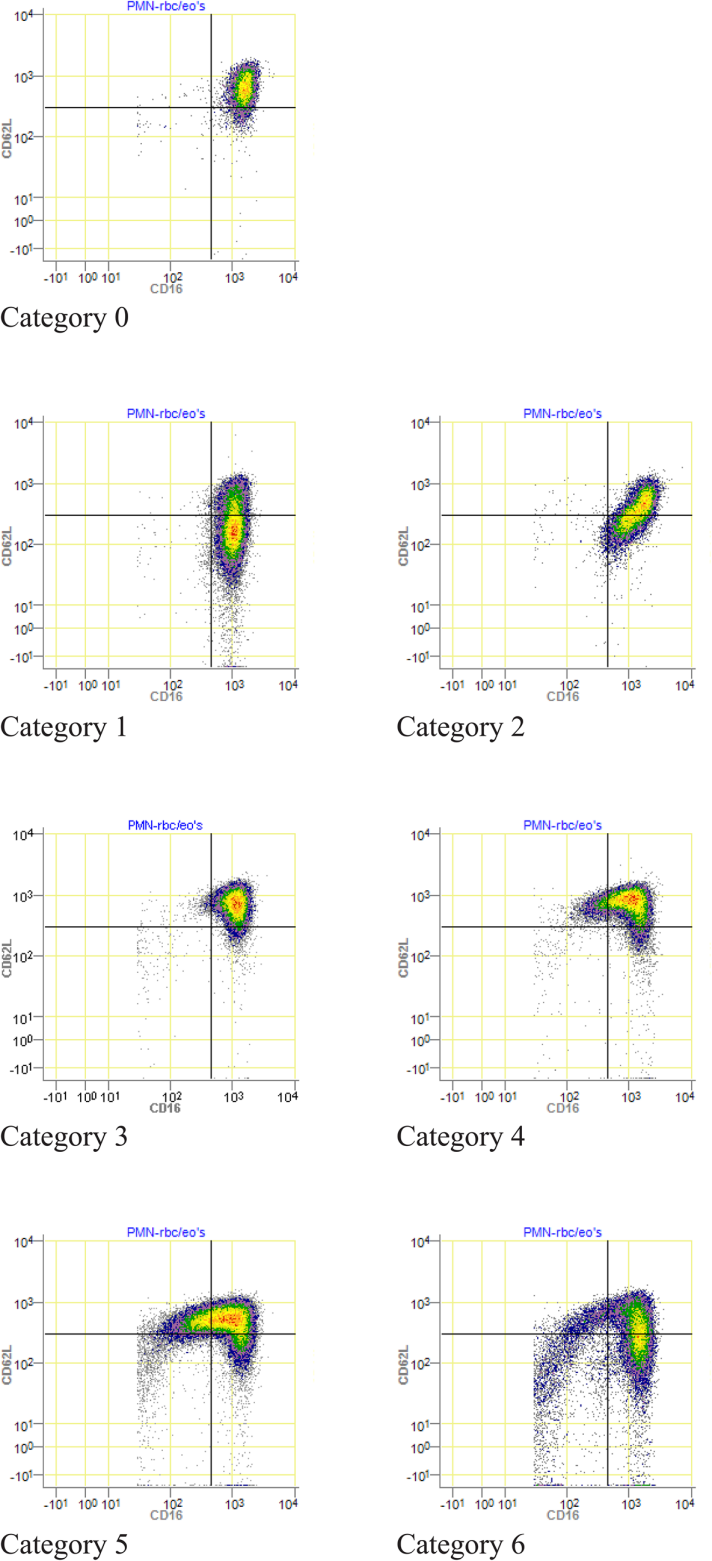
Representative individual patient samples to illustrate the immunophenotype categories based on the occurrence of subsets of neutrophils in CD16/CD62L dot plots

Seven immunophenotype categories were identified. As shown in Fig. 2, Category 0 consists of no subsets, Category 1 only shows a (larger) CD62L^dim^ subset, and Category 2 consists of one mixed subset in the lower left quadrant of the dot plot, where the expression of both CD16 and CD62L tends to be negative. Category 3 consists of two small (only purple dots) and similar in size CD16^dim^ and CD62L^dim^ subsets, Category 4 consists of bigger (green and yellow dots, representing more cells) CD16^dim^ and CD62L^dim^ subsets, and Category 5 shows extensive CD16^dim^ and CD62L^dim^ subsets, where the CD16^dim^ subset is bigger than the CD62L^dim^ subset. Category 6 shows an immunophenotype of mainly neutrophil progenitors, as well as CD62L^dim^ neutrophils. All included patient samples were divided into one of the immunophenotype categories. Tables 2 and 3 shows that for the statistical analysis, Category 5 and Category 6 were merged into one group (Category 5+) because of the low number of patients in Category 5 and Category 6 and because both categories consist of the most extreme neutrophil subsets (CD16^dim^/banded neutrophils and progenitors). Category 5+ consisted of 10 patients. All patients in category 5+ were significantly injured. The two patients in Category 6 died within 12 h. All included individual patient samples are displayed in Online Resource 3. In addition, individual healthy control samples of CD16/CD62L expression are shown in Online Resource 4.

**Table 2:**
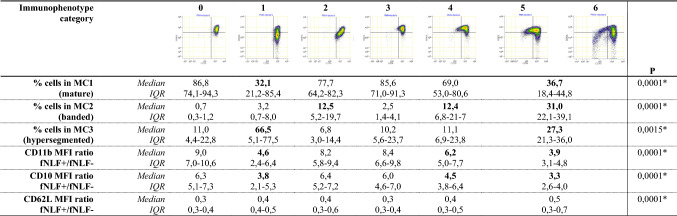
Infographic of the distribution of neutrophil populations and responsiveness to fNLF as analyzed by FlowSOM in immunophenotype categories after trauma

Category 5 and 6 merged into one Category 5+ . fNLF± with and without fNLF stimulation. *p* values of Kruskal–Wallis *H* test displayed. Significant *p* values indicated with *. Categories with significant differences compared to Category 0 after post hoc testing indicated with bold

*MC* metacluster, *MFI* mean fluorescent intensity, *fNLF* *N*-formyl-norleucyl-leucyl-phenylalanine

**Table 3:**
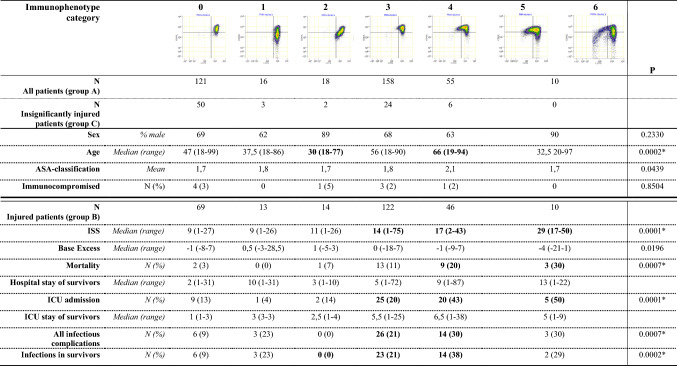
Infographic of patient/injury characteristics and clinical outcome in immunophenotype categories after trauma

Category 5 and 6 merged into one Category 5+

*p* values of Kruskal–Wallis *H* test displayed. Significant *p* values indicated with *. Categories with significant differences compared to Category 0 after post hoc testing indicated with bold

*ISS* injury severity score, *ICU* intensive care unit

### FlowSOM analysis and immunophenotype categories

Table 2 shows that the immunophenotype categories differ significantly in terms of percentage of cells in each metacluster and in terms of responsiveness to fNLF.

The percentage of mature neutrophils (MC 1) is lower in Categories 2, 4 and 5+ compared to Category 0 (*p* < 0.0001). The percentage of banded neutrophils (MC 2) is higher in each Category compared to Category 0 (*p* < 0.0001), in Category 2 and 4 the median percentages banded cells increases to respectively 12.5% and 12.4%. In Category 5+ the median percentage banded cells increases even up to 31%. The percentage of hypersegmented neutrophils (MC 3) increases in Category 1 and Category 5+ (*p* < 0.0015). The ratio MFI fNLF+/fNLF− of CD11b and CD10 decreases in Category 1, 4 and 5+ (*p* < 0.0001).

### Immunophenotype categories and patient characteristics

Eighty-five patients had no significant injuries that needed admittance to the hospital and were analyzed as a reference group (group C). Most patients were in the ASA 1 or 2 category. Nine patients were immunocompromised which was defined by the use of immunocompromising medicine (e.g., Methotrexate, Infliximab, Cellcept), HIV infection or Common Variable Immunodeficiency Disorder (CVID). The immunocompromised patients did not display significant differences in the numbers of neutrophil subsets, nor did any other investigated results differ for this group. Immunocompromised patients were, therefore, not excluded from further analysis.

In group A, there was a statistically significant difference in age between patients in different categories (*p* < 0.0002). Post hoc analysis revealed differences between immunophenotype categories: patients in Category 2 were younger and patients in Category 4 were older than patients in other categories (*p* < 0.006) (see Table 3, Online Resource 5).

Furthermore, there was no significant relationship between sex, ASA-classification, or immunocompromised patients and immunophenotype category after trauma (see Table 3).

### Immunophenotype categories and injury characteristics

Trauma mechanisms of included patients consisted of road traffic injuries, falls, penetrating trauma, blunt trauma, shooting/stab incidents and drowning, causing injuries of any severity in any body region (monotrauma, multitrauma, head and neck injuries, chest trauma, abdomen trauma). Patients admitted to the hospital had an Injury Severity Score (ISS) between 1 and 75 (mean 14.58 ± SD 11.14, median 13, range 1–75, IQR 8–19). An increasing injury severity was seen in Category 3–6, indicating more severe inflammation in case of more severe injury: *χ*^2^(5) = 41.114, *p* = 0.000. Injury was significantly less severe in Category 0–2 (see Table 3, Online Resource 6).

### Clinical outcome

As shown in Table 4, the mean duration of in-hospital stay was 8.3 days, and the mean duration of ICU stay was 5.5 days. None of the included patients had an acute infection requiring antibiotics at the time of presentation at the trauma bay, or were considered acutely ill on admission. During hospital admission 52 of 275 (19%) patients developed infectious complications. The most reported infectious complications were pneumonia, wound infections, urinary tract infections, secondary meningitis, line infections, and fracture-related infections. Twenty-eight patients died in the hospital, of which one death was infection related. ISS was significantly higher (*p* < 0.001) in infectious patients (median 17, range 1–75, IQR 11–26) compared to non-infectious patients (median 11, range 1–75, IQR 6–17). The development of infectious complications was significantly different between patients in different immunophenotype categories (*p* < 0.001). The percentage of patients that developed infectious complications was higher in Category 4 and 5+ (30%), than in categories 0, 1, 2 and 3 (9–21%) (see Table 3).

**Table 4:** Overview of clinical outcomes of all included trauma patients (group A) and of all trauma patients admitted at the UMCU during the year 2020

	All patients (group A) *N* (%)	Annual trauma patients (2020) *N* (%)
Total trauma team activations	380	± 1800
Hospital admission	295 (78)	1243 (69)
Hospital stay (days)		
Mean ± SD Median (range)	8.9 ± 12.2 4 (1–87)	5.8
IQR	1–12	
ICU admission	62 (16)	258 (14)
ICU stay (days)		
Mean ± SD	6.9 ± 8.2	1
Median (range) IQR	3 (1–38) 1–9	
Infectious complication	52 (14)	NR
In hospital death	28 (8)	80 (4)

Patients who died in the hospital were excluded for analysis of hospital stay and ICU stay. Statistical significance could not be determined due to a lack of data

## Discussion

Immunophenotype categories based on visual pattern recognition of neutrophil subsets, as measured in a fully automated flow cytometer, are feasible and easily clinical applicable to provide insight into the inflammatory response immediately after trauma. Cluster analysis, performed using FlowSOM, showed that these predefined categories are valuable, as the amount of banded (MC2) and hypersegmented (MC3) neutrophils and the responsiveness to fNLF differed significantly between the different categories (see Table 2). This supports the concept that visual interpretation of 2D FACS dot plots holds valuable information about different neutrophil populations. Visual interpretation can easily be done by a clinician and can hold valuable clinical information about the immune status of trauma patients. However, it would be preferable to analyze visual information in a more objective manner, e.g., by artificial intelligence or with machine learning programs. This could not yet be achieved and requires further research with much larger numbers to make this clinically applicable. On the other hand, the use of these visual categories is a first approach that is useful to interpret flow cytometry results after trauma by the clinician directly at the bedside, in the same manner as interpreting e.g., an X-ray or CT scan.

Our results demonstrated that Categories 3–6 were each related to a more severe level of injury and a greater risk of infectious complications after trauma. This is in line with previous studies based on CD16^dim^ neutrophils and its clinical consequences (i.e., mortality, hospital stay) [15]. Furthermore, the outcomes of Category 6 confirm previous findings that this is an extreme immunophenotype that occurs only in patients in extremis (e.g., severe shock, traumatic resuscitation, full drowning) and is associated with minimal chance of survival.

As no relation was found between the patient characteristics independent of trauma and the immunophenotype Categories 3–6, it is likely that the appearance of these cells is a direct consequence of the inflammatory response initiated by tissue damage, after an injury-severity threshold is reached. This finding reinforces the fact that the impact of injuries and the subsequent tissue damage determines the inflammatory response, which can be visualized in a fast and non-biased way at the bedside by the characterization of neutrophil subsets in the circulation. Measuring the immunophenotype of severely injured patients immediately after trauma might be the next step to improve personalized and point-of-care decision making in trauma care. Moreover, the course of the inflammatory response is dynamic in time and is driven by both patient characteristics, injury type and severity, timing and extent of surgery. Further determination of these changes over time will increasingly facilitate personalized care of injured patients. As it is now possible to measure the neutrophil functional phenotype at any time point after trauma, the longitudinal course of the inflammatory response and the impact of additional interventions can be followed. In the future, measuring the neutrophil compartment over time could possibly guide decision making regarding timing and extent of immune protective surgery and determine the administration of (preventive) antibiotics [22].

Second, our results showed that patients in Categories 0–2 had similarly low injury severity scores. Thus, it is tempting to speculate that patients in Categories 0–2 display immune profiles that are not significantly initiated by tissue damage and the subsequent release of DAMPs into the circulation. These patients may display such immune phenotype intrinsically, regardless of any injury. Supporting this hypothesis is the finding that, even though the differences were not significant, patients in Category 1 seemed to be more prone to infectious complications than patients in Category 0 and Category 2 (see Table 3). The patient and injury characteristics give no explanation for the different outcome in this group. Further research is warranted into such putative personal immune profiles or individualized immune responses to limited inflammatory stimuli and the factors influencing them. Such inherent profiles can be studied in patients who have completely recovered from their trauma, as ‘before-measurements’ are impossible in trauma patients. If these immune profiles prove to provide insight in a personal immune response and a subsequent risk of infectious complications, it would be of value to assess the immunophenotype for every trauma patient presented at the trauma bay with suspected injuries that require full diagnostic work up.

The patients in Category 1 (with CD62L^dim^ cells) showed a decreased responsiveness to fNLF and seemed more prone to develop infectious complications compared to patients in Category 0, 2 and 3 (see Table 2). Previous studies demonstrated a relation between a decreased responsiveness towards fNLF immediately after trauma and severe sepsis after a week [23]. In addition, the subset of neutrophils characteristic for Category 1 (CD62L^dim^) was found to be less capable of preventing bacterial outgrowth because of impaired intracellular bacterial containment [14]. Together with the refractoriness to stimulation with formyl-peptides, this can lead to a clinical situation that is associated with a higher risk of infectious complications after trauma [16]. The reason why the patients in Category 1 display this phenotype of immune cells remains to be identified.

The immunophenotype found in patients in Category 2 represents a subset of neutrophils that has not yet been described in literature, nor was it noticed in our previous research. Our data demonstrated that patients in this category were younger than patients in the other categories, and none developed infectious complications. Although the small sample size of this category should be kept in mind, this might suggest the presence of a neutrophil subset with a different functionality. It may be that these cells express other activation markers than CD16/CD62L that could give us further information about the cells. The neutrophil activation marker CD11b was analyzed, but no difference in expression of this marker between the immunophenotype categories was observed. To gain more insight into the subset of neutrophils in Category 2, these cells should be sorted, and functional assays must be performed to assess whether or not they show differences in functionality as well. Moreover, the cytospins of these cells should be made to obtain more information about the morphology of these cells. However, these measurements were beyond the scope of this current project.

Earlier studies suggested that neutrophil subtypes are involved in the pathogenesis of infectious complications after trauma [7, 14, 23, 24]. Understanding the functions and characteristics of neutrophil subtypes is important to understand their involvement in the development of infectious complications. Our results demonstrated that patients with extended neutrophil subsets in their blood after trauma were more severely injured and had a higher risk of infectious complications. Prior research described CD16^dim^ neutrophils as an immature neutrophil population with a higher bacterial containment capacity than other neutrophil subsets [25]. This supports the hypothesis that trauma can lead to massive mobilization of the best functioning (CD16^dim^) neutrophils from the bone marrow. However, this might lead to a subsequent imbalance of the neutrophil compartment when this process takes too long, as the bone marrow will be unable to fully compensate in time for the loss of these well-functioning neutrophils [26]. This might lead to a relatively high sensitivity to infectious complications [27]. A second explanation is the partial desensitization of the bone marrow to subsequent stimuli such as intruding (bacterial) pathogens after the first hit of major injury and the recruitment of young neutrophils [20]. In previous research it was shown that this type of immunoparalysis could be simulated in a two-challenge LPS endotoxemia model, where indeed fewer CD16^dim^ cells were recruited after a second challenge [28]. This mechanism might also be of relevance in trauma patients suffering from infectious complications.

One of the objectives of this project was to determine if the immunophenotype categories correlated with pre-existent patient and injury characteristics. One of the patient characteristics that possibly determine the inflammatory response is age. Earlier research described elevated plasma concentrations of neutrophil stimulating cytokines in a healthy elderly population, contributing to an enhanced inflammatory status created by continuous stimulation of the innate immune system generally referred to as inflamm-ageing [29]. This could lead to a lower capacity of the immune system to liberate the correct numbers of the different neutrophil subsets during acute inflammation. This is in line with studies showing an age-related decrease in phagocytic- and bactericidal capacity in neutrophils [30]. However, in this study no significant differences in immunophenotype categories in the elderly population were observed. Further research into the neutrophil compartment of the elderly trauma patient should be conducted in a larger study population to further investigate these changes in the aging immune system. Furthermore, it could be hypothesized that different types of injury (penetrating or blunt) or even injury in different body regions (e.g., thorax trauma, isolated brain injury or a fractured femur) could lead to a different amplitude of inflammatory reaction to the tissue damage. This was previously shown in a population with either blunt or penetrating thorax trauma [31]. As for the elderly population, further research should be conducted into the reaction of the immune system to different types of injuries in a larger prospective cohort.

This study is unique in the fact that a large amount of reproducible flow data were generated by the easy-to-use point-of-care “load&go” flow cytometry directly after trauma. The studied trauma population is very heterogenic, containing a wide range of severity of injuries. The approach of including all patients admitted at the trauma bay, regardless of injury severity, enabled the analysis of a broad spectrum of patient characteristics in the context of the inflammatory response. ISS was not used as an inclusion criterion, because ISS is a poor predictor of tissue damage, poorly reflects physiological challenges such as shock a drowning, and possibly underestimates the burden of injury due to tissue damage, which is better reflected in the inflammatory response. Furthermore, ISS is calculated mostly after several days. Our data show that, although patients who develop infectious complications have a significantly higher ISS, there is a wide range of outliers, from ISS 1 to ISS 75. This indicated that the ISS did not perform well as a specific predictor for risk of infectious complications. Our hypothesis was that individual patients display an individual inflammatory profile as a response to trauma. However, no correlation was found between host-dependent variables and inflammatory response to trauma. A limitation of this retrospective cohort is that the study population represents the more severely injured patients admitted at the trauma bay of a level one trauma center such as the UMC Utrecht. The included population had a longer in-hospital and intensive care unit stay and the percentage of patients who died in the hospital was higher in the included patients compared to all trauma patients admitted at the UMCU during the year 2020 (see Table 4). These differences can be explained by the exclusion of all patients under the age of 18 years and due to the fact that drawing blood samples is less necessary in patient without severe injuries.

In conclusion, the distribution of neutrophil subsets described in phenotype categories is valuable and easily recognizable for clinicians at the bedside. No correlation was found between patient-specific variables (e.g., age, sex, comorbidity) and inflammatory response as described by neutrophil phenotype categories, indicating that the investigated patient characteristics did not contribute to a specific inflammatory profile. After a certain threshold, injury severity was related to the immunophenotype Categories 3–6. Furthermore, patients in higher categories had a significantly higher risk of developing infectious complications. This interpretation of the immunophenotype of a patient immediately after trauma is a fast and non-biased way of visualizing injury severity and the subsequent inflammatory response at the bedside, which might be the next step to improving personalized and point-of-care decision-making in trauma care.

## Supplementary Information

Below is the link to the electronic supplementary material.

Supplementary file1 Supplementary Material 1 STROBE Checklist (PDF 146 KB)

Supplementary file2 Supplementary Material 2 Representing examples of neutrophil populations clustered by FlowSOM (PDF 479 KB)

Supplementary file3 Supplementary Material 3 All included individual patient samples (CD16/CD62L dot plots) divided into 7 immunophenotype categories based on the occurrence of subsets of neutrophils in CD16/CD62L dot plots (PDF 6194 KB)

Supplementary file4 Supplementary Material 4 Healthy control samples displaying dot plots of baseline neutrophil CD16/CD62L expression (PDF 679 KB)

Supplementary file5 Supplementary Material 5 Age (years) of all included patients (group A) in different immunophenotype categories. All flow cytometry measurements executed on admission, during diagnostic work up in the trauma bay (PDF 118 KB)

Supplementary file6 Supplementary Material 6 Injury severity score (ISS) of all injured patients (group B) in different immunophenotype categories. All flow cytometry measurements executed on admission, during diagnostic work up in the trauma bay (PDF 109 KB)
