# Supplementary material for: Visualization of the inflammatory response to injury by neutrophil phenotype categories: Neutrophil phenotypes after trauma
Source: Eur J Trauma Emerg Surg. 2022 Nov 8;49(2):1023–34. doi: 10.1007/s00068-022-02134-3 (PMC10175373; doi:10.1007/s00068-022-02134-3)
Supplement: Supplementary file 2 — Supplementary file2 Supplementary Material 2 Representing examples of neutrophil populations clustered by FlowSOM (PDF 479 KB) [file 68_2022_2134_MOESM2_ESM.pdf]

Supplementary Information

Article title: Visualization of the inflammatory response to injury by neutrophil phenotype categories

Journal name: European Journal of Trauma and Emergency Surgery

Author names: E.J. de Fraiture, S.H. Bongers, L. Koenderman, N. Vrisekoop, K.J.P. van Wessem, L.P.H. Leenen, F. Hietbrink

Corresponding author: F. Hietbrink MD/PhD, Department of Trauma Surgery, University Medical Center Utrecht, The Netherlands. E-mail address: f.hietbrink@umcutrecht.nl

Representing example of overlaid dot plot of neutrophil population clustered by FlowSOM in metacluster 1, 2 and 3:

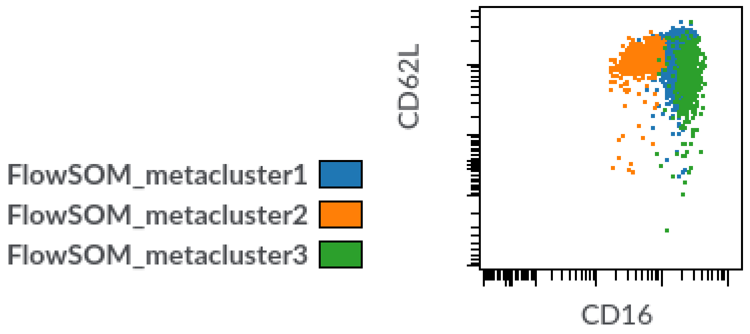

Representing examples of FlowSOM analysis for each immunophenotype category:

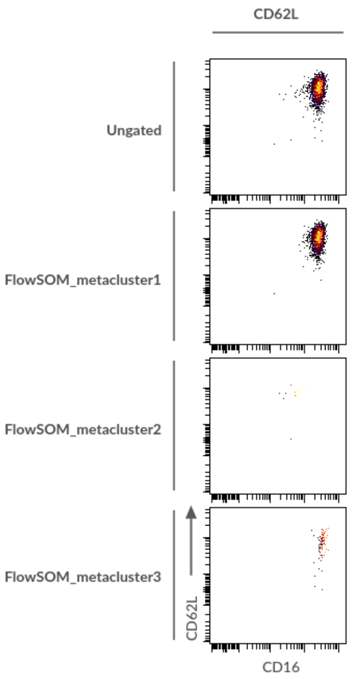

Category 0

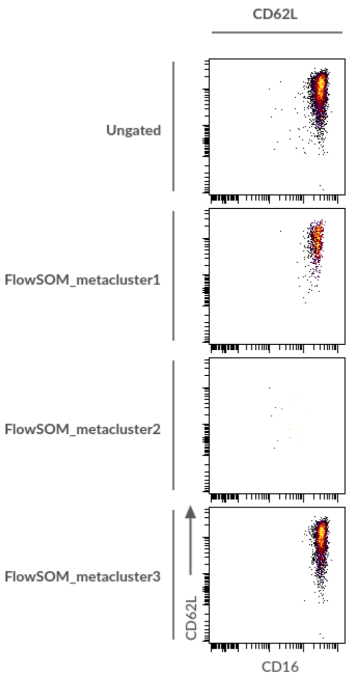

Category 1

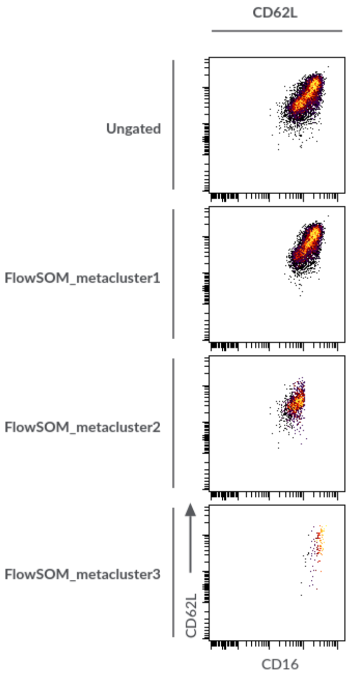

Category 2

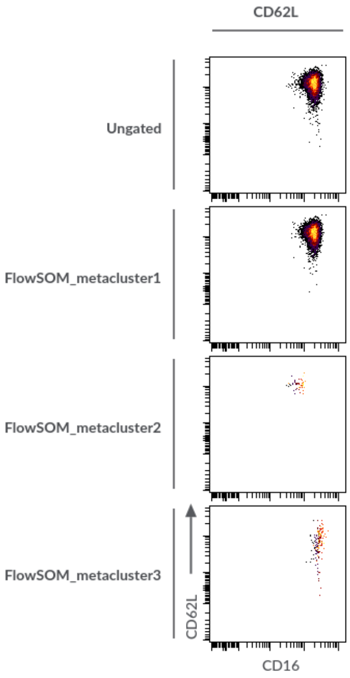

Category 3

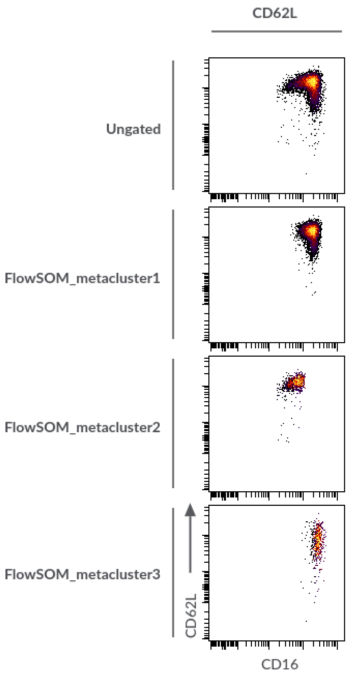

Category 4

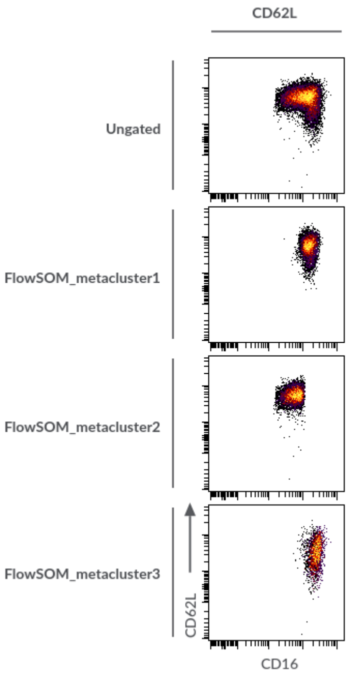

Category 5

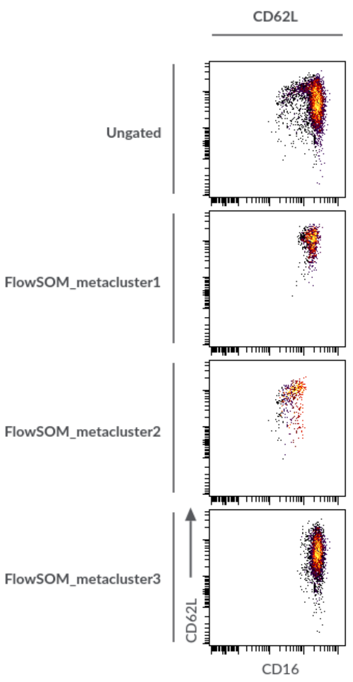
CD62L
Ungated
FlowSOM_metacluster1
FlowSOM_metacluster2
FlowSOM_metacluster3
CD62L
CD16

Category 6
