# Supplementary material for: Visualization of the inflammatory response to injury by neutrophil phenotype categories: Neutrophil phenotypes after trauma
Source: Eur J Trauma Emerg Surg. 2022 Nov 8;49(2):1023–34. doi: 10.1007/s00068-022-02134-3 (PMC10175373; doi:10.1007/s00068-022-02134-3)

**Supplementary Information**

Article title: Visualization of the inflammatory response to injury by neutrophil phenotype categories

Journal name: European Journal of Trauma and Emergency Surgery

Author names: E.J. de Fraiture, S.H. Bongers, L. Koenderman, N. Vrisekoop, K.J.P. van Wessem, L.P.H. Leenen, F. Hietbrink

Corresponding author: F. Hietbrink MD/PhD, Department of Trauma Surgery, University Medical Center Utrecht, The Netherlands. E-mail address: f.hietbrink@umcutrecht.nl

## Category 0

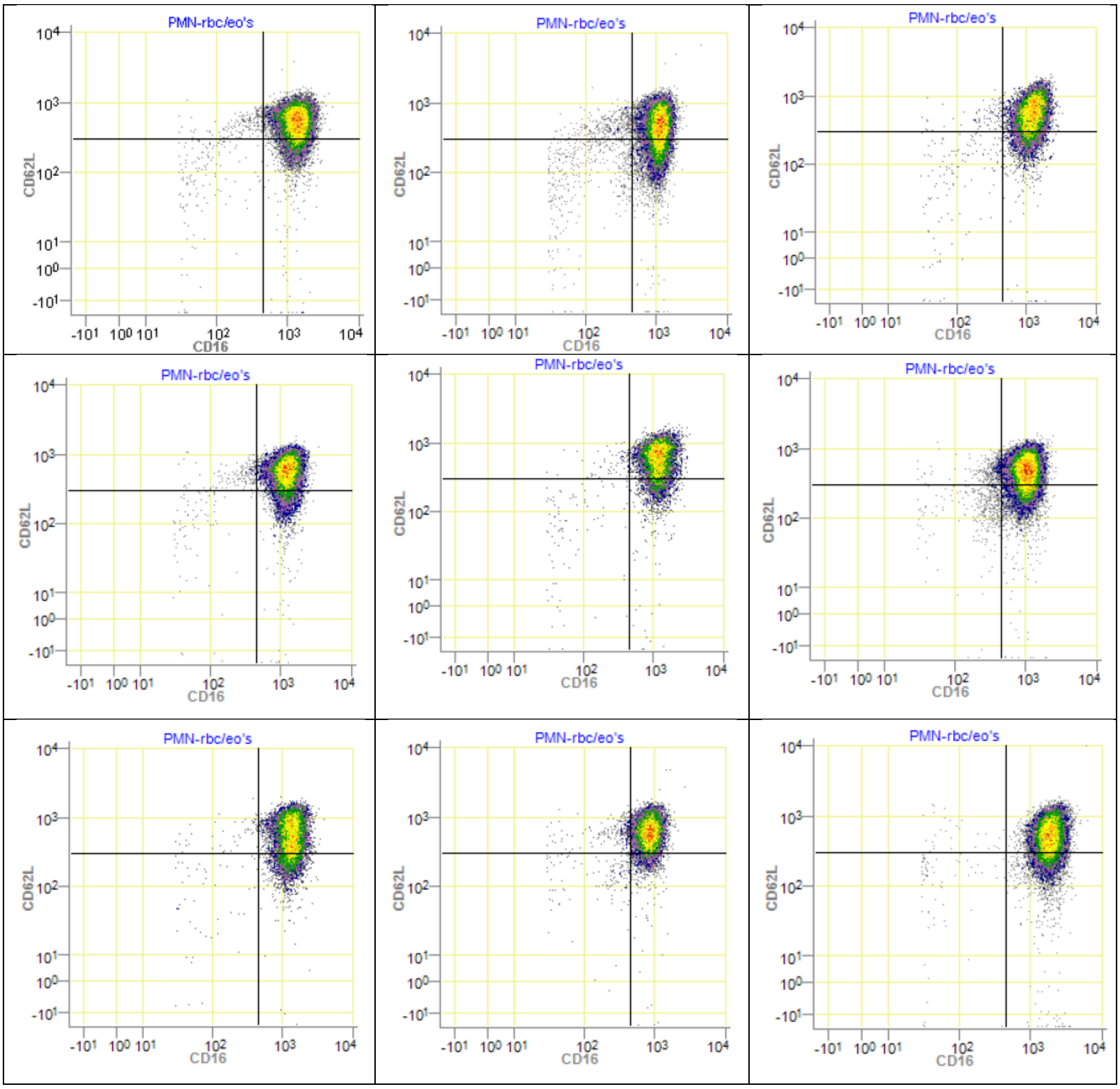

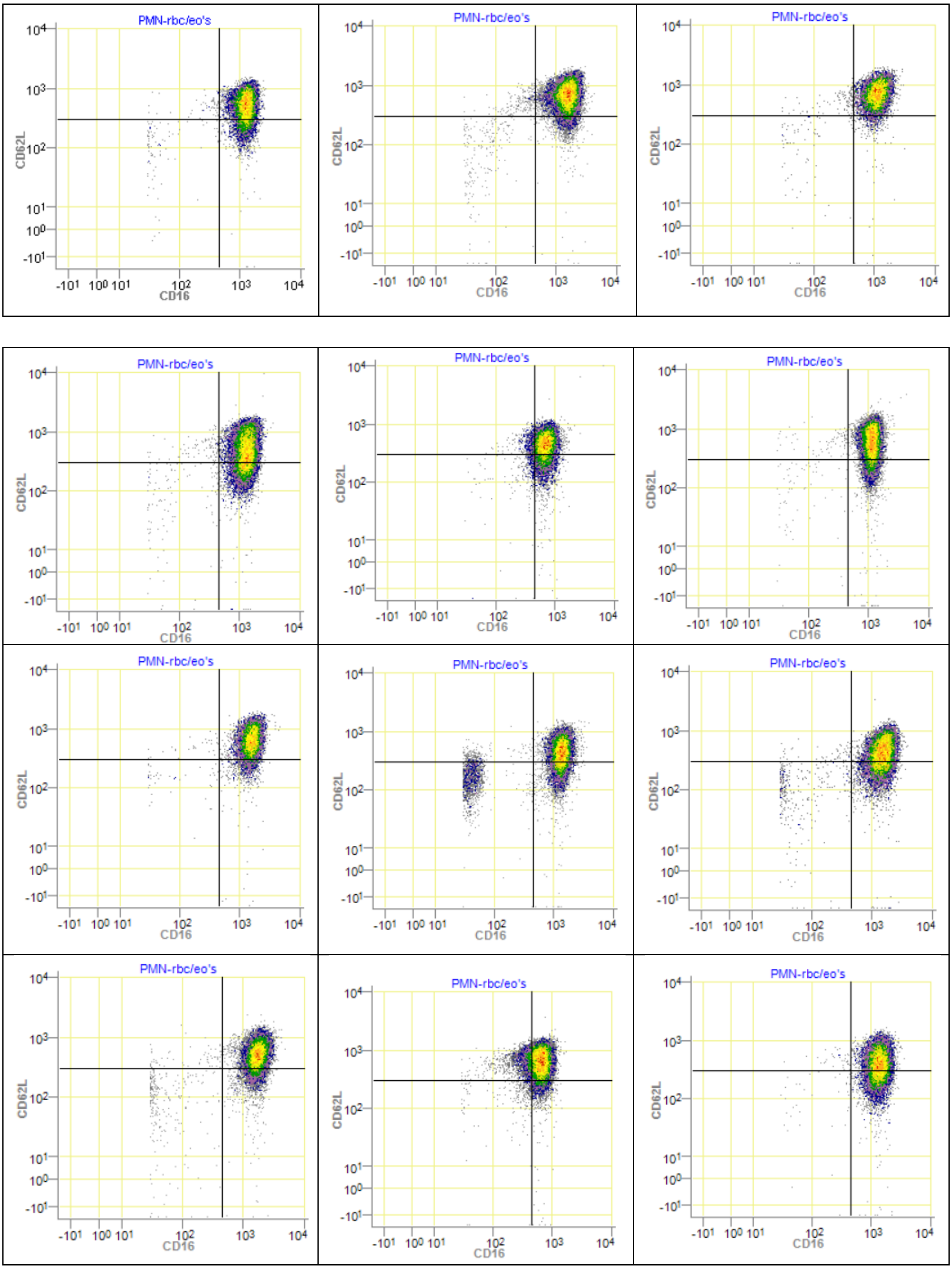

PMN-rbc/eo's
CD62L
CD16
$10^4$
$10^3$
$10^2$
$10^1$
$10^0$
$-10^1$

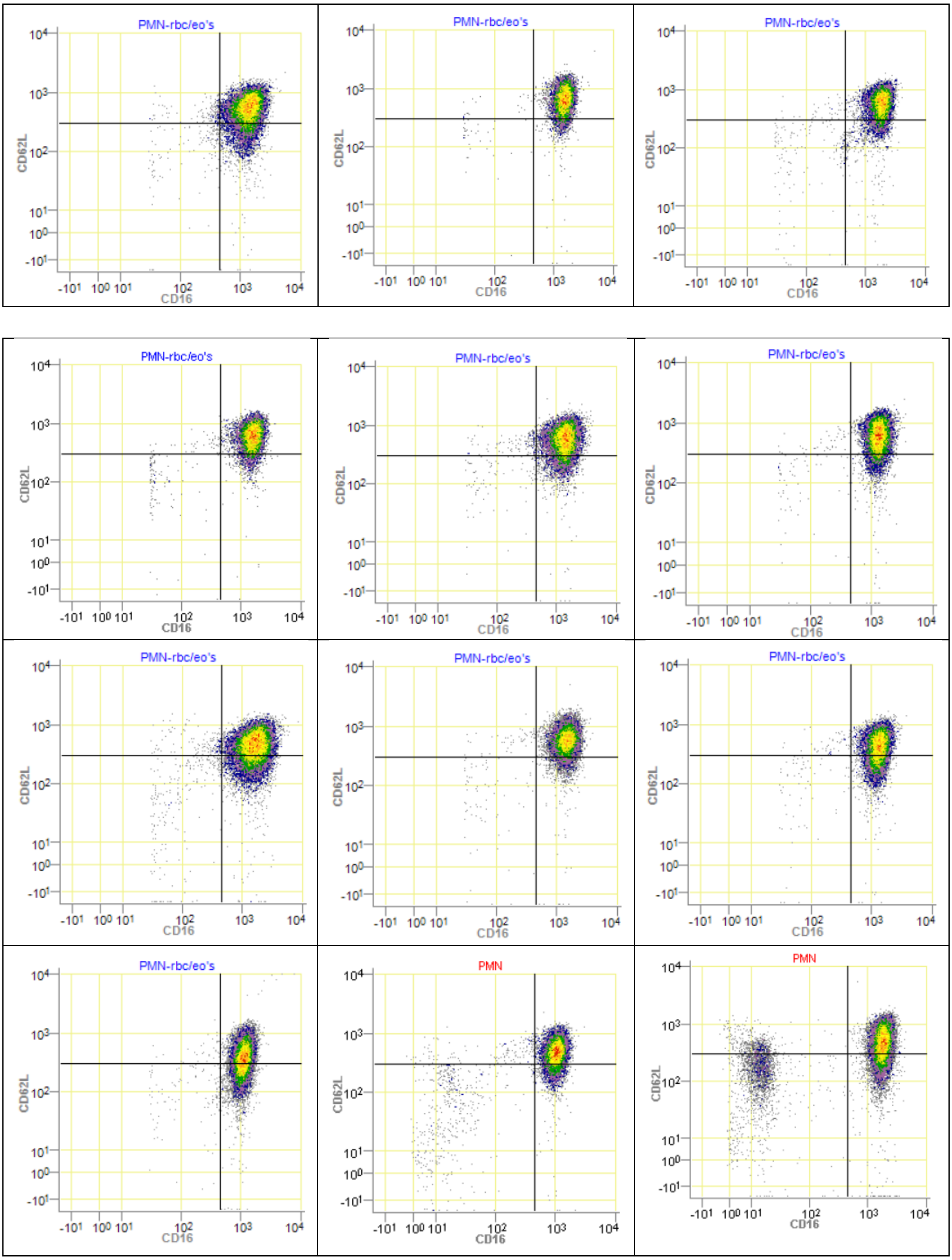
PMN-rbc/eo's
PMN-rbc/eo's
PMN-rbc/eo's
PMN-rbc/eo's
PMN-rbc/eo's
PMN-rbc/eo's
PMN-rbc/eo's
PMN-rbc/eo's
PMN-rbc/eo's
PMN-rbc/eo's
PMN
PMN
CD62L
CD16

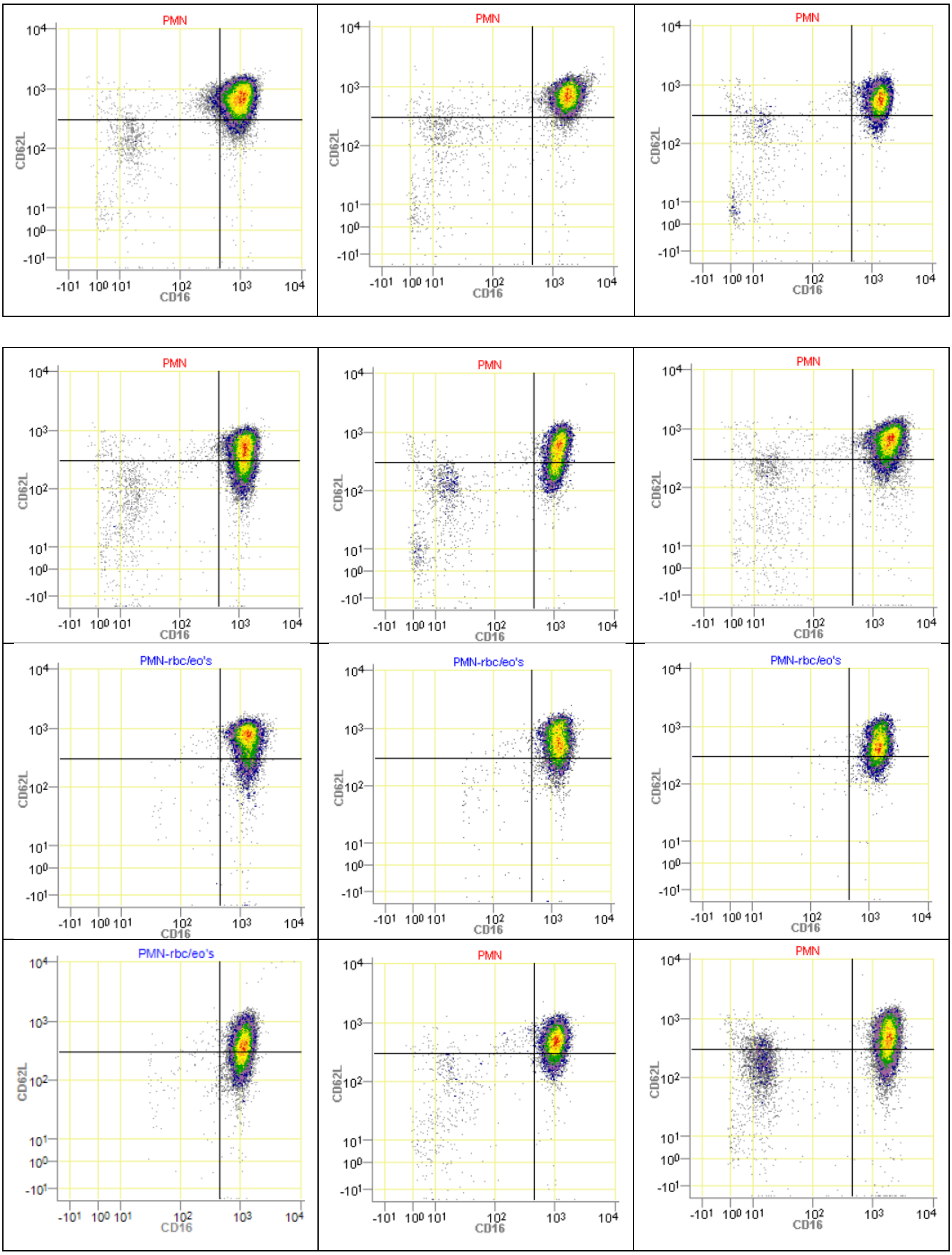
PMN
PMN
PMN
PMN
PMN
PMN
PMN-rbc/eo's
PMN-rbc/eo's
PMN-rbc/eo's
PMN-rbc/eo's
PMN
PMN
CD62L
CD16

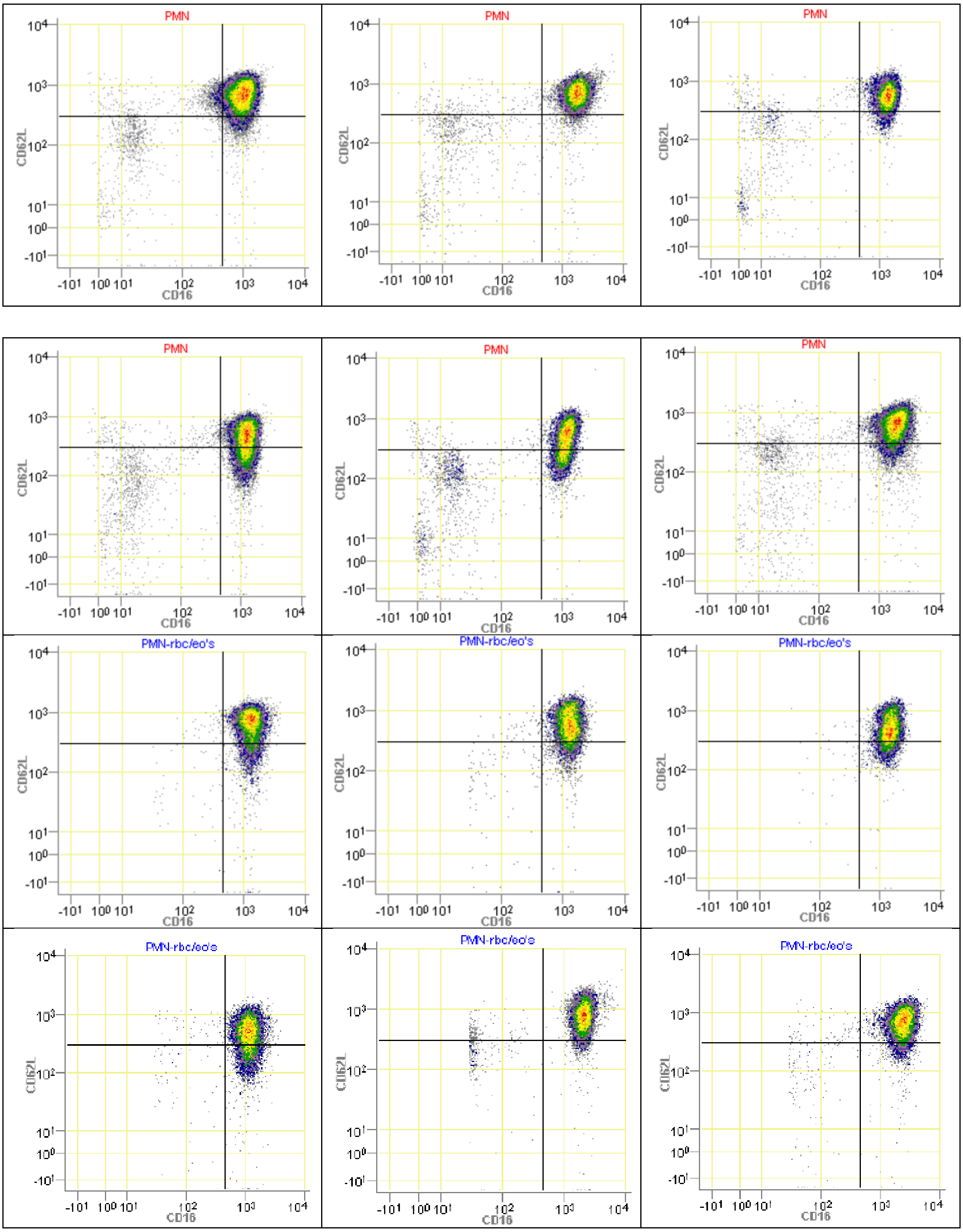
PMN
PMN
PMN
PMN
PMN
PMN
PMN-rbc/eo's
PMN-rbc/eo's
PMN-rbc/eo's
PMN-rbc/eo's
PMN-rbc/eo's
PMN-rbc/eo's
CD62L
CD16
$10^4$
$10^3$
$10^2$
$10^1$
$10^0$
$-10^1$

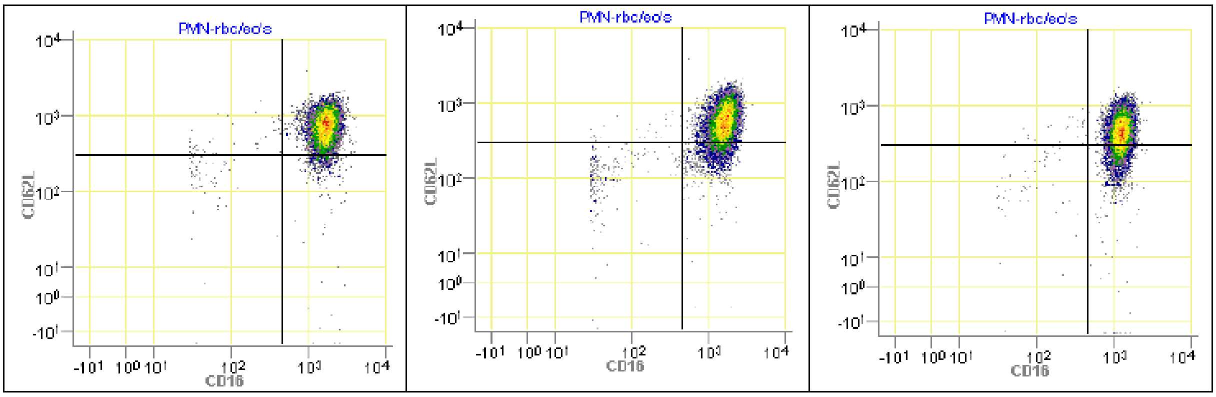
PMN-rbc/eo's
CD62L
CD16
104
103
102
101
100
-101


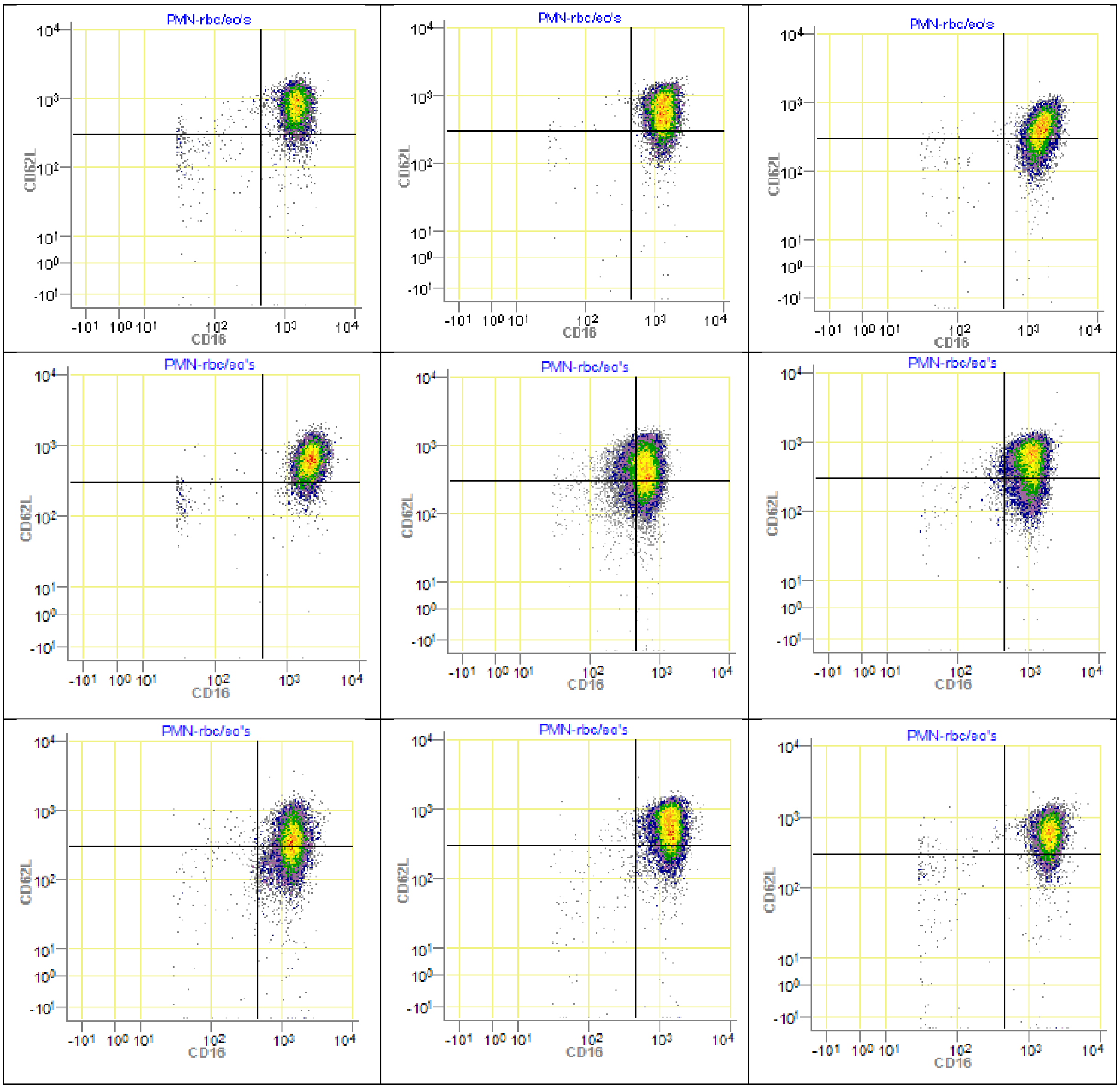
PMN-rbc/eo's
CD62L
CD16
104
103
102
101
100
-101

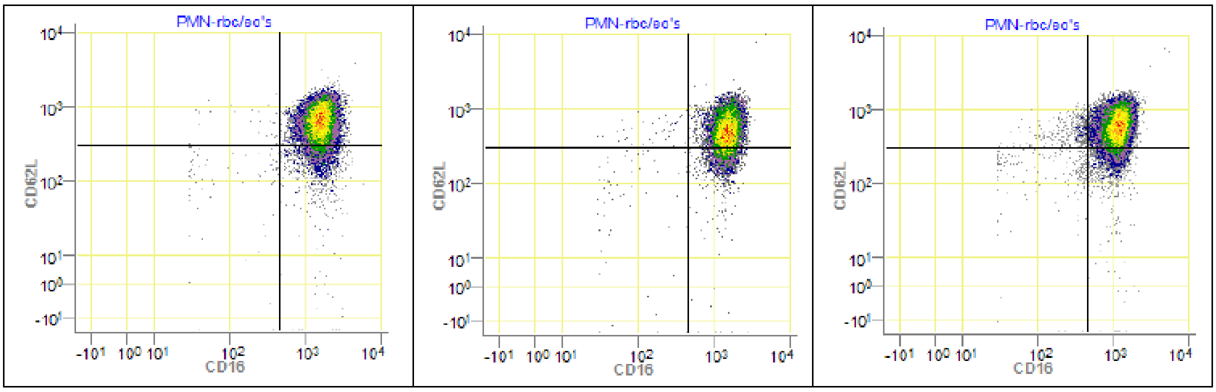
PMN-rbc/eo's
CD62L
CD16
10⁴
10³
10²
10¹
10⁰
-10¹

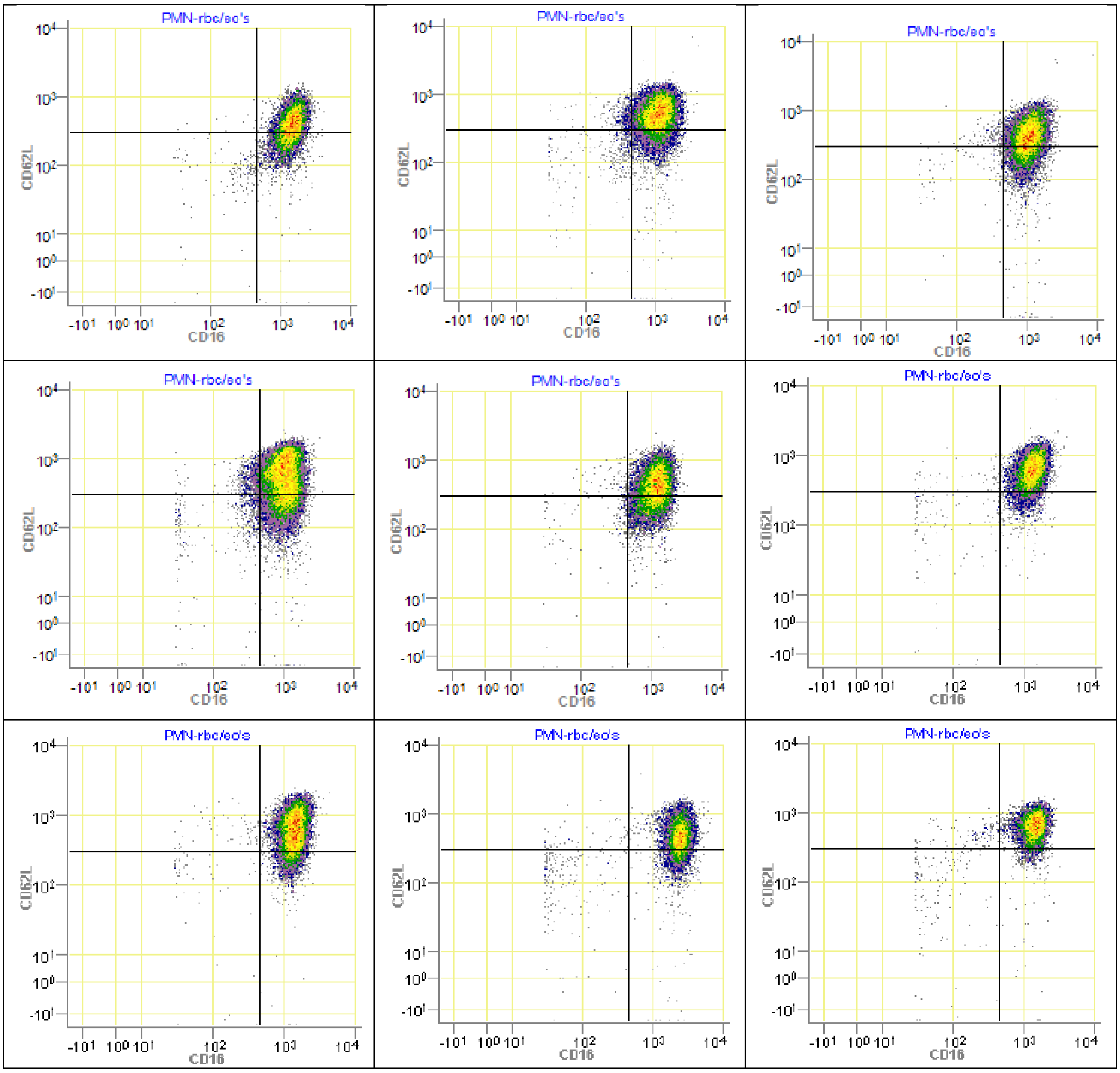
PMN-rbc/eo's
CD62L
CD16
10⁴
10³
10²
10¹
10⁰
-10¹

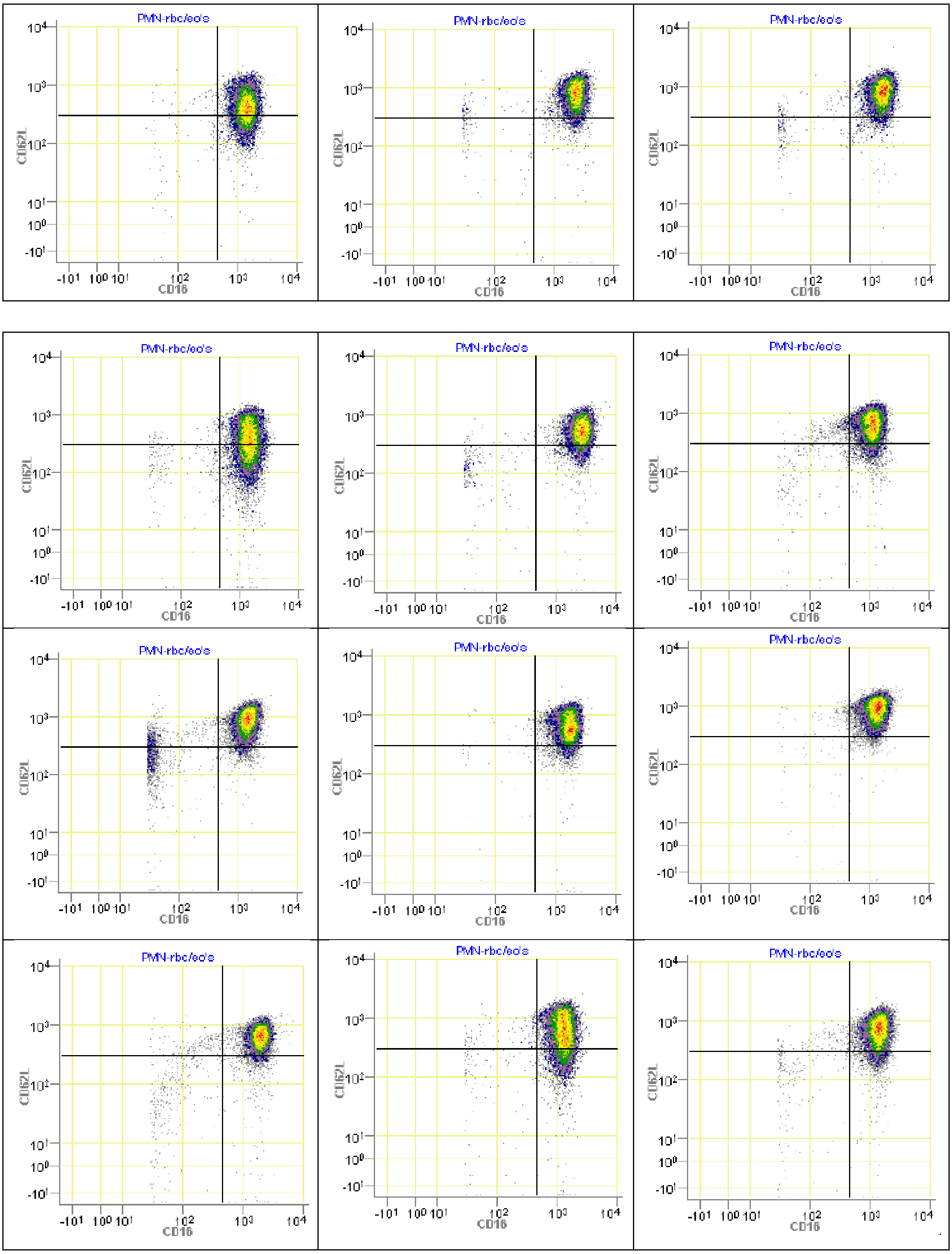
PMN-rbc/eo's
CD62L
CD16
10^4
10^3
10^2
10^1
10^0
-10^1
-10^1 10^0 10^1 10^2 10^3 10^4

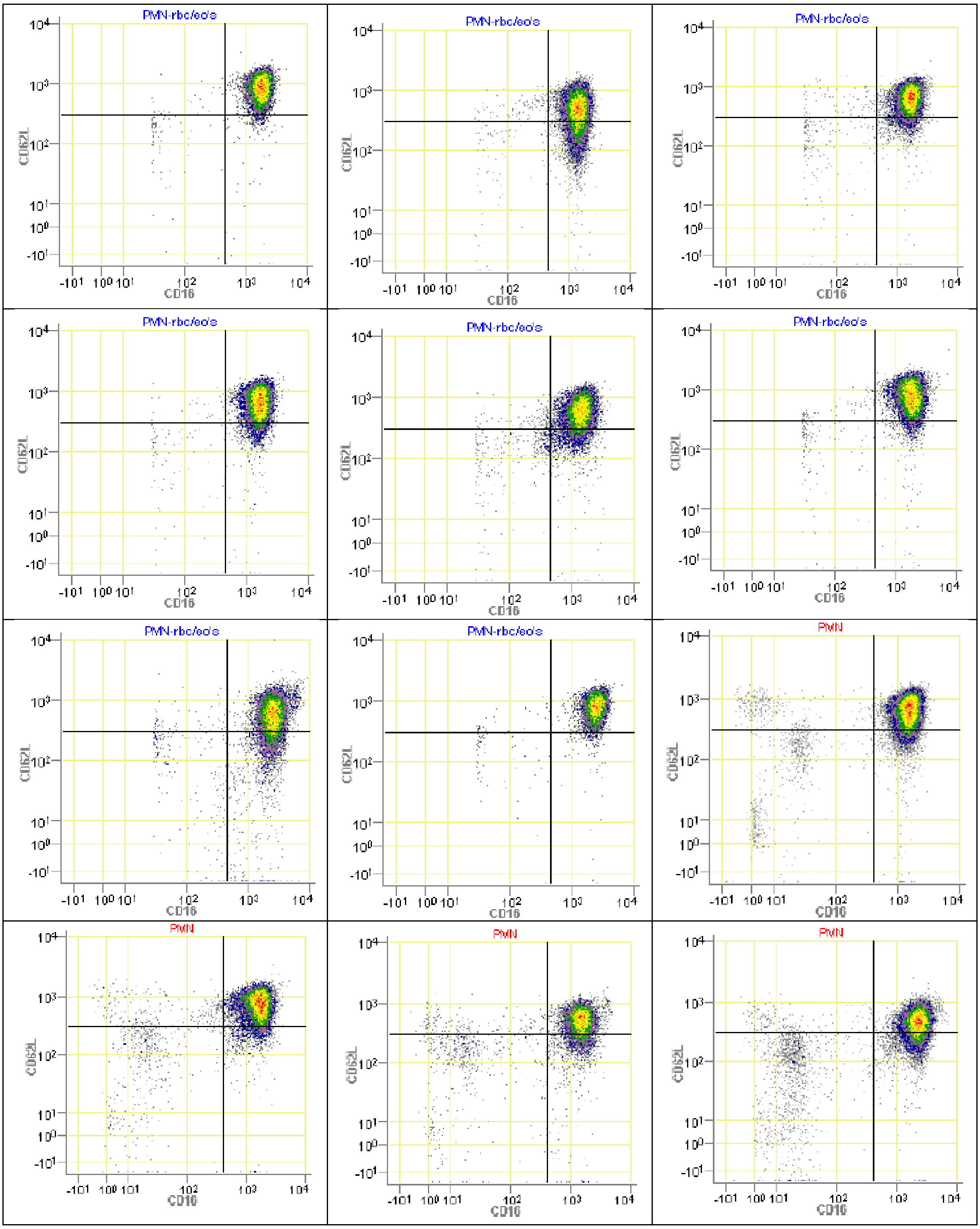
PMN-rbc/eo's
PMN-rbc/eo's
PMN-rbc/eo's
PMN-rbc/eo's
PMN-rbc/eo's
PMN-rbc/eo's
PMN-rbc/eo's
PMN-rbc/eo's
PMN
PMN
PMN
PMN
CD62L
CD16

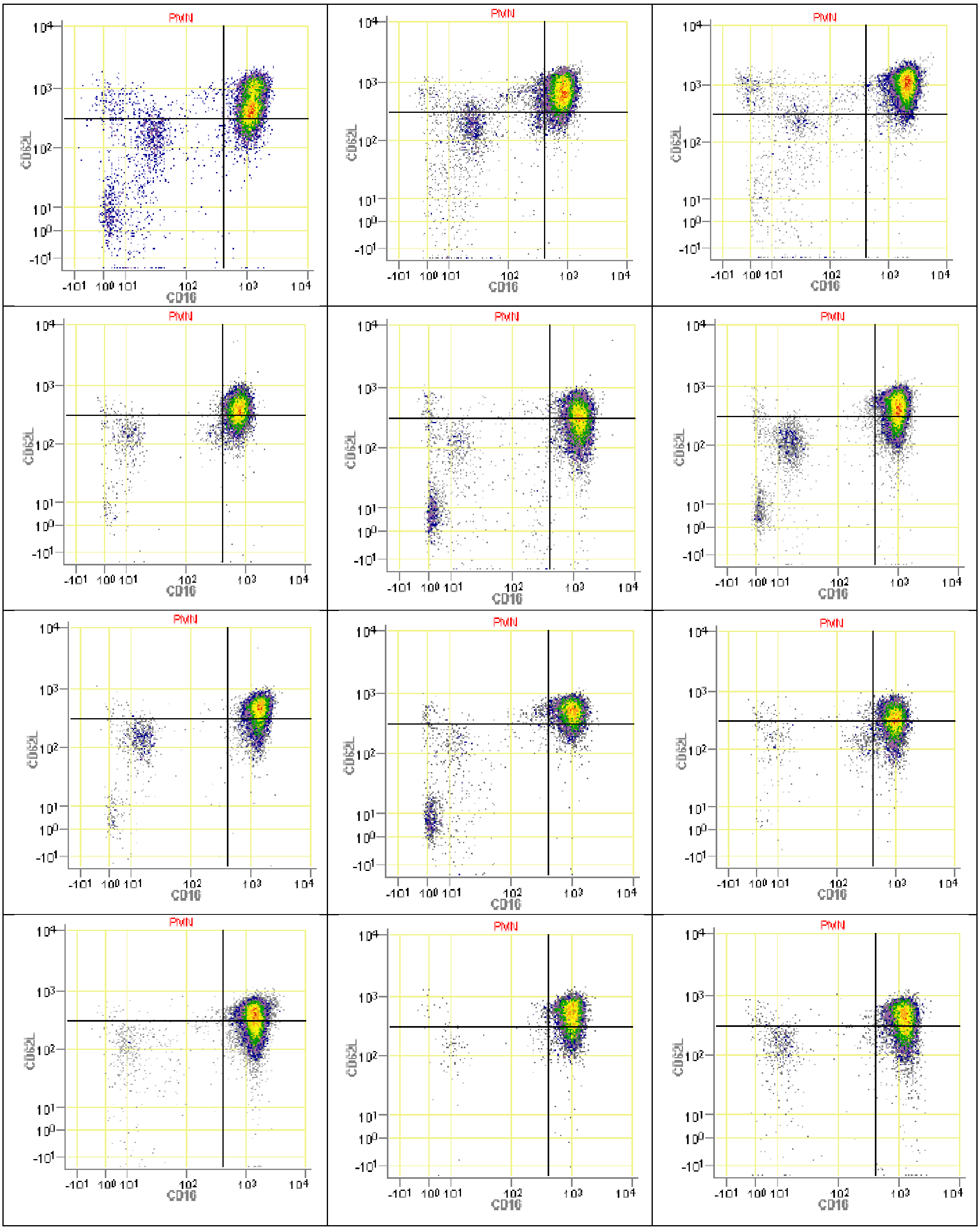
PMN
CD62L
CD16
$10^4$
$10^3$
$10^2$
$10^1$
$10^0$
$-10^1$

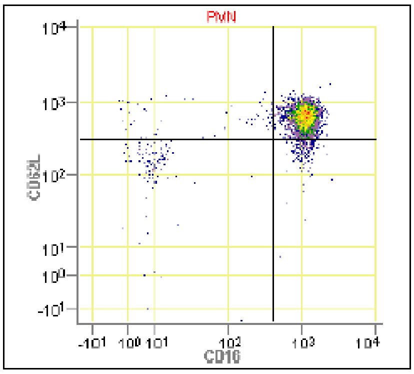

PMN
CD62L
CD16

# Category 1

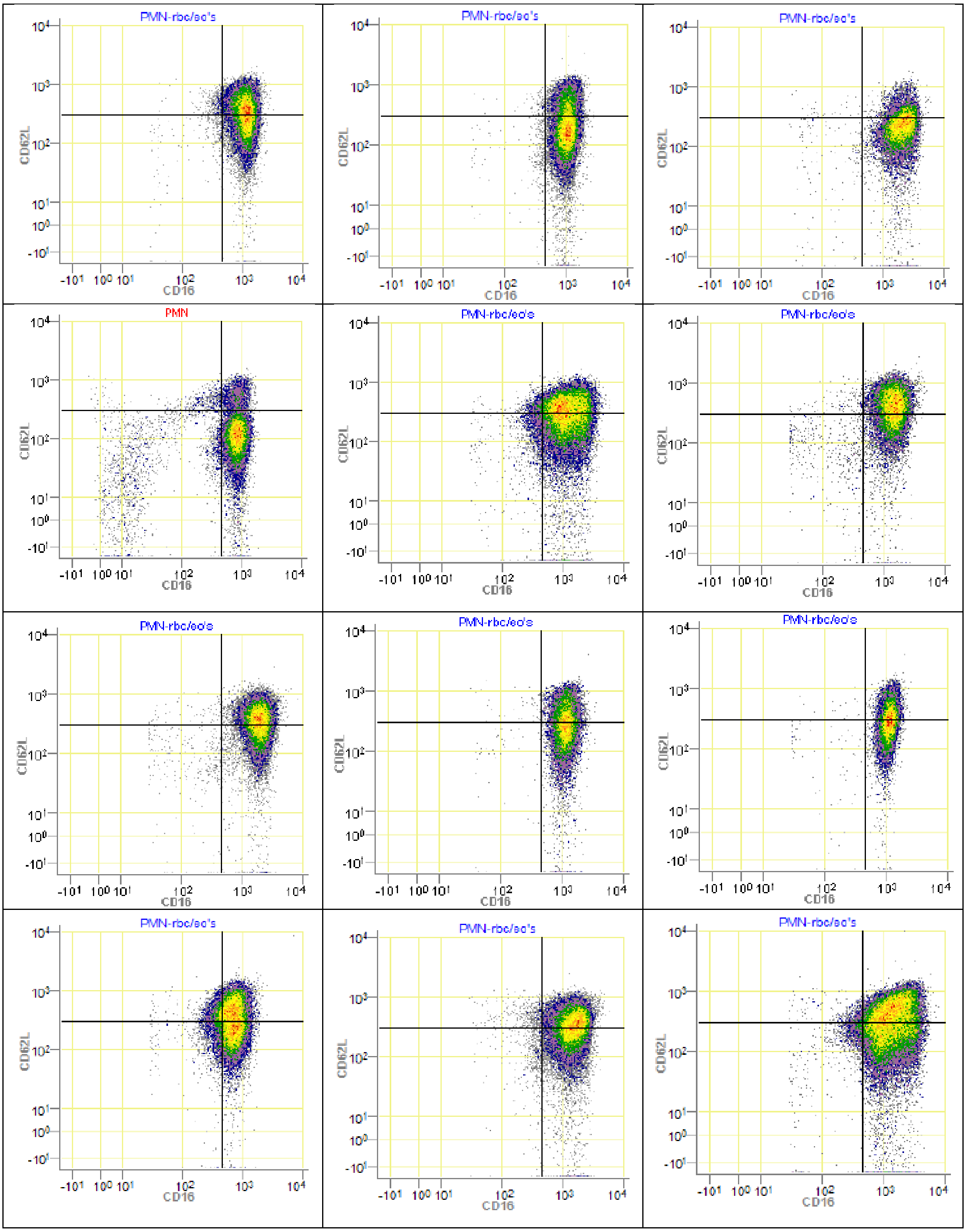
PMN-rbc/eo's
PMN-rbc/eo's
PMN-rbc/eo's
PMN
PMN-rbc/eo's
PMN-rbc/eo's
PMN-rbc/eo's
PMN-rbc/eo's
PMN-rbc/eo's
PMN-rbc/eo's
PMN-rbc/eo's
PMN-rbc/eo's
CD62L
CD16
-10¹ 10⁰ 10¹ 10² 10³ 10⁴

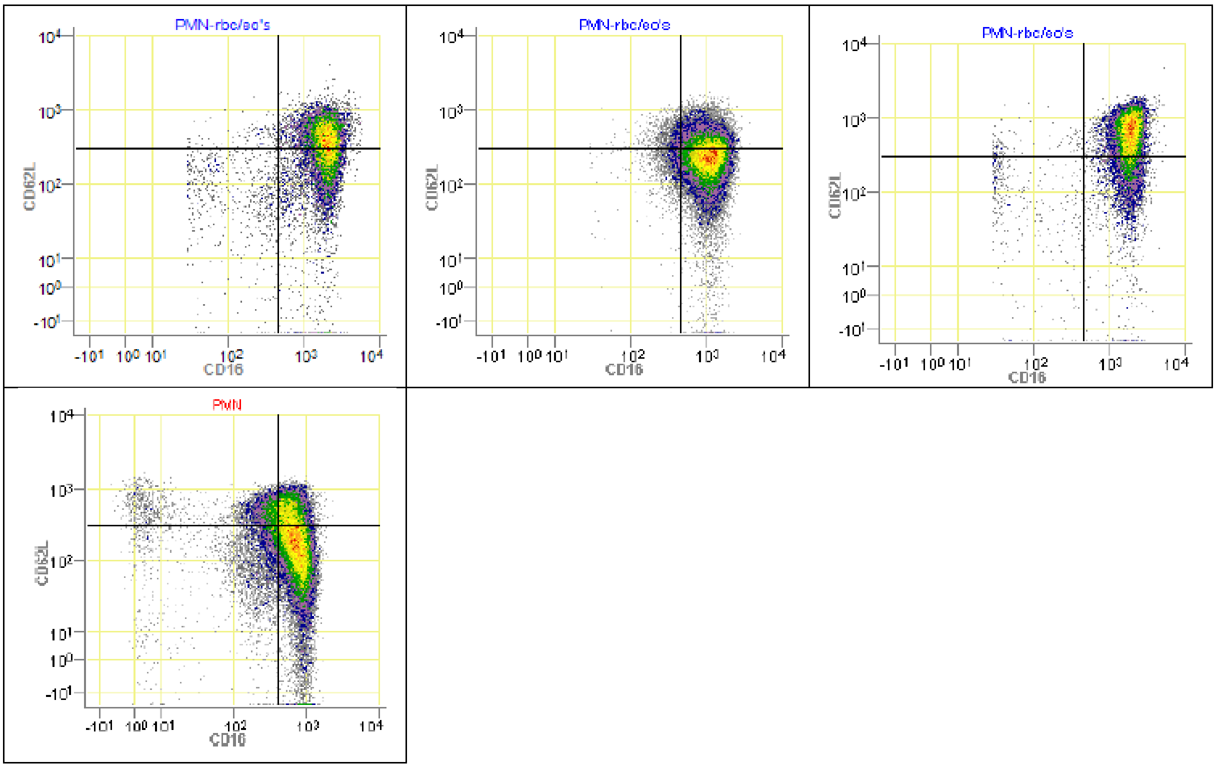
PMN-rbc/eo's
CD62L
CD16
PMN-rbc/eo's
CD62L
CD16
PMN-rbc/eo's
CD62L
CD16
PMN
CD62L
CD16

# Category 2

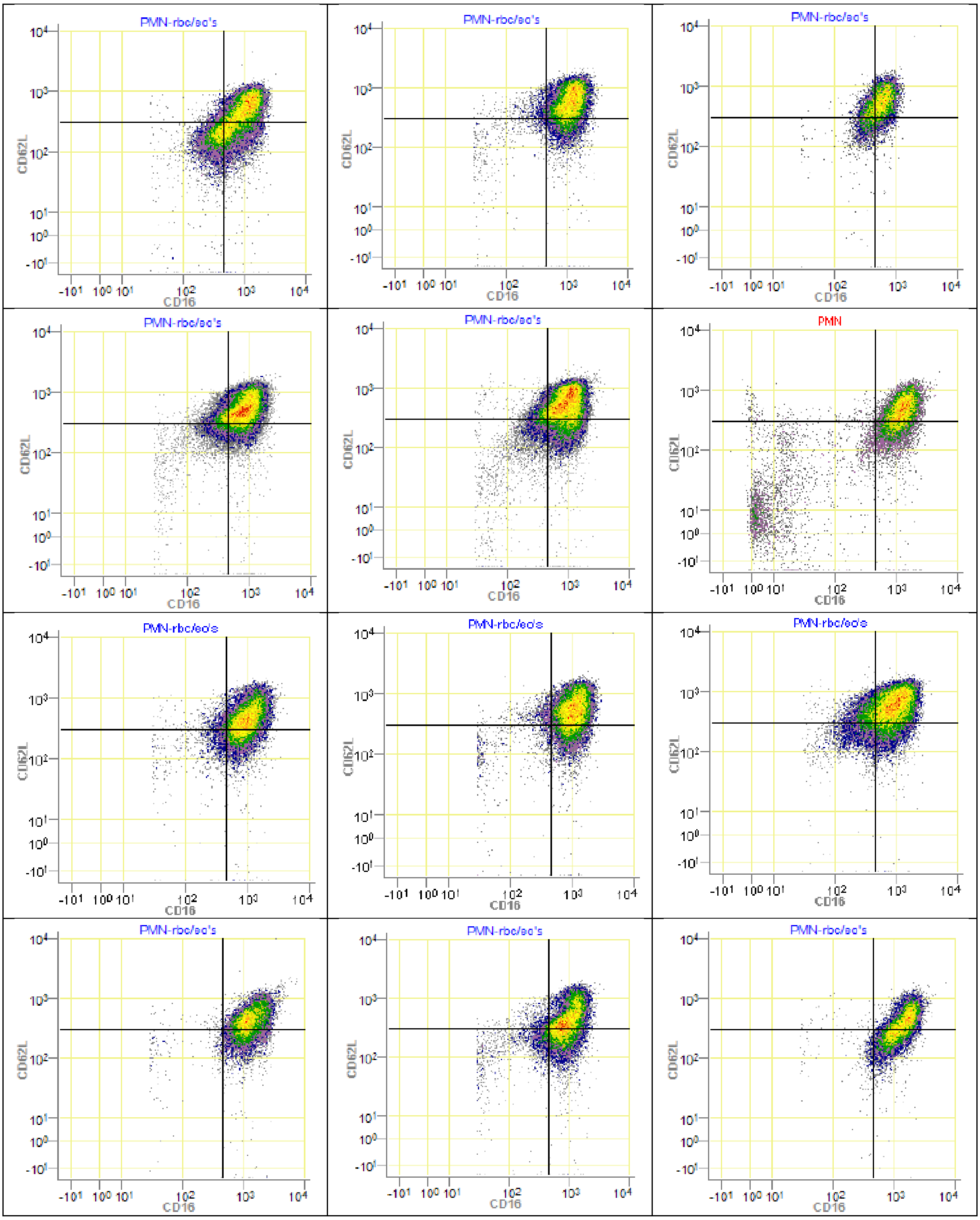
PMN-rbc/eo's
PMN-rbc/eo's
PMN-rbc/eo's
PMN-rbc/eo's
PMN-rbc/eo's
PMN
PMN-rbc/eo's
PMN-rbc/eo's
PMN-rbc/eo's
PMN-rbc/eo's
PMN-rbc/eo's
PMN-rbc/eo's
CD62L
CD16

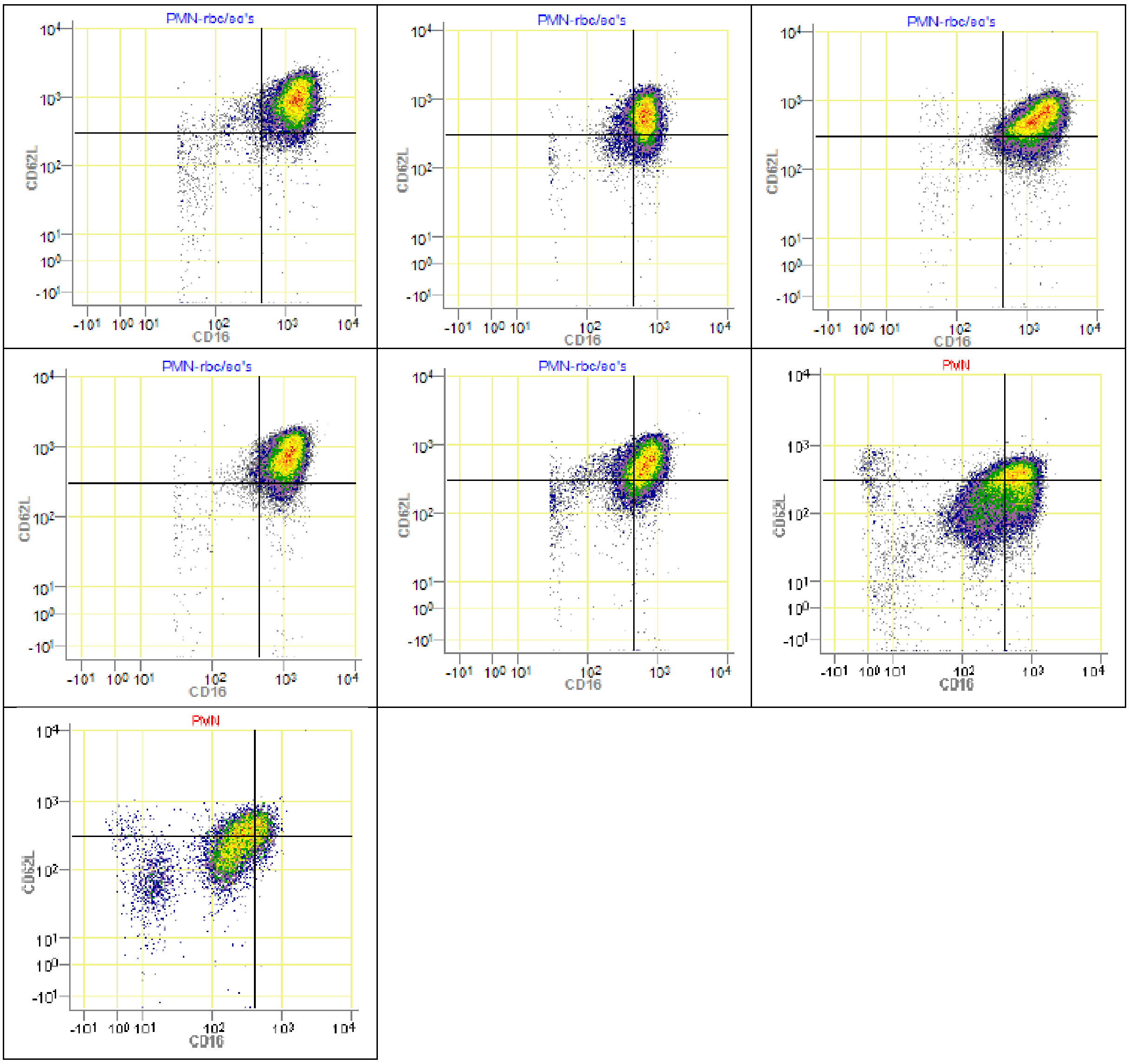
PMN-rbc/ec's
CD62L
CD16
PMN-rbc/ec's
CD62L
CD16
PMN-rbc/ec's
CD62L
CD16
PMN-rbc/ec's
CD62L
CD16
PMN-rbc/ec's
CD62L
CD16
PMN
CD62L
CD16
PMN
CD62L
CD16

# Category 3

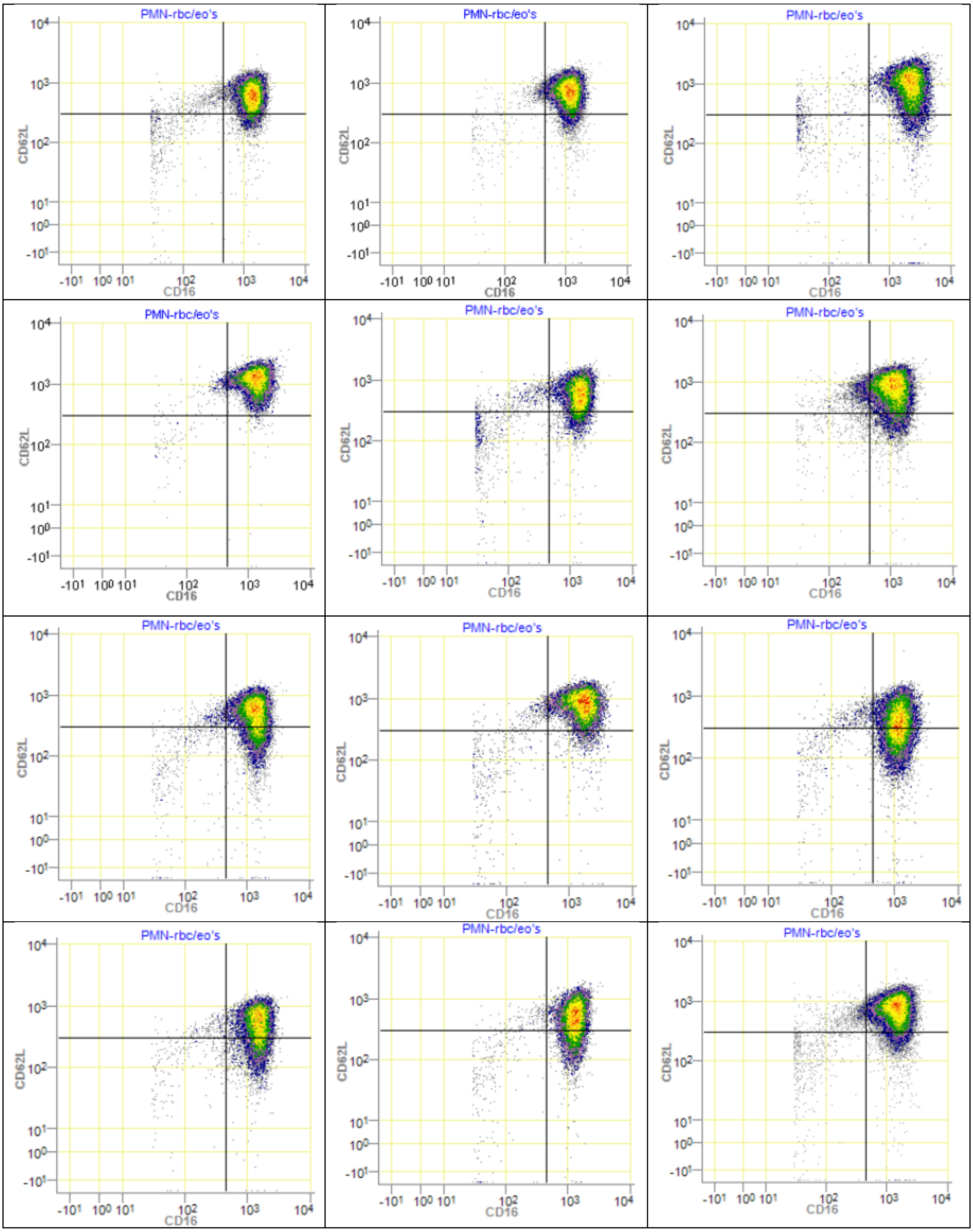

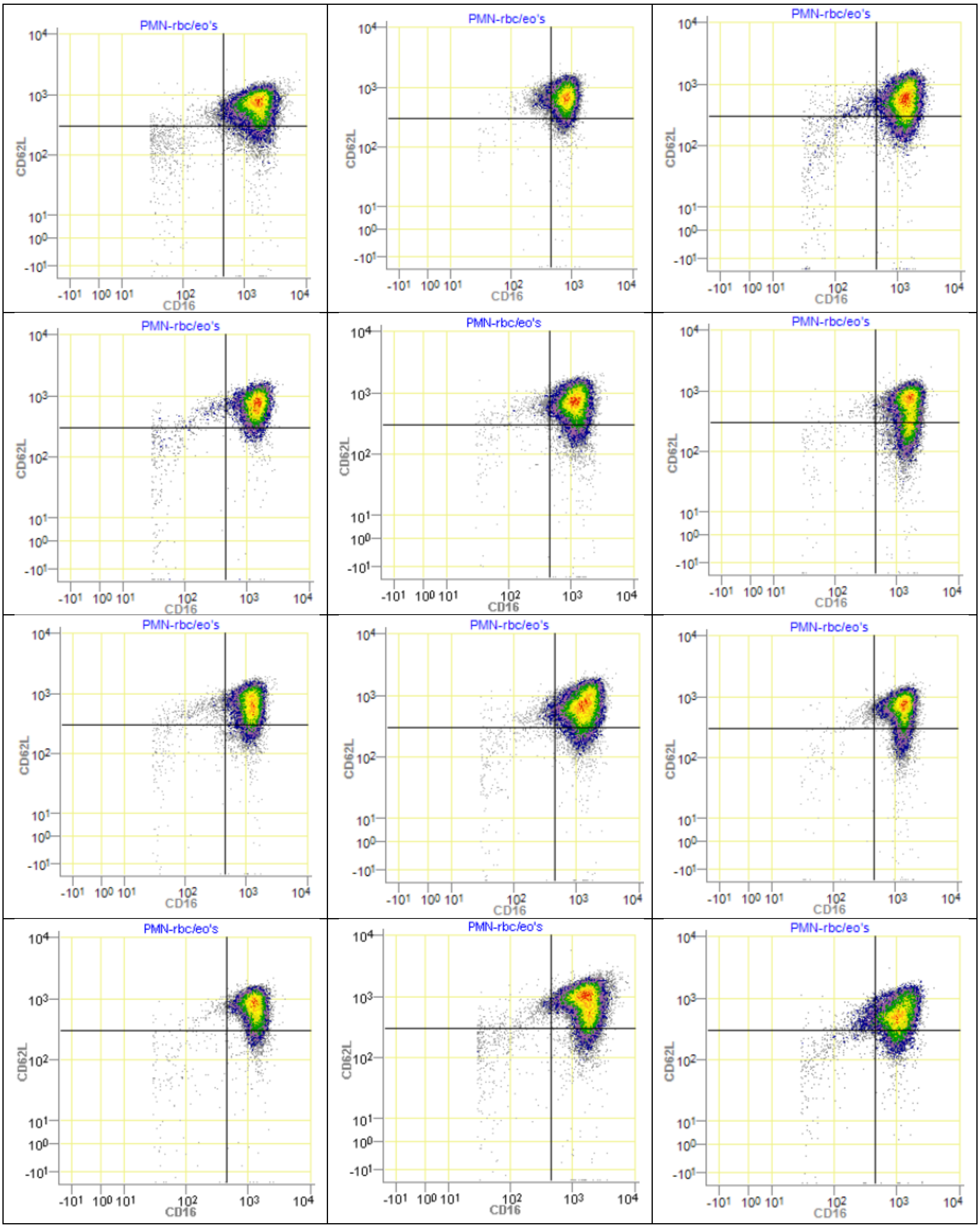
PMN-rbc/eo's
CD62L
CD16
$10^4$
$10^3$
$10^2$
$10^1$
$10^0$
$-10^1$

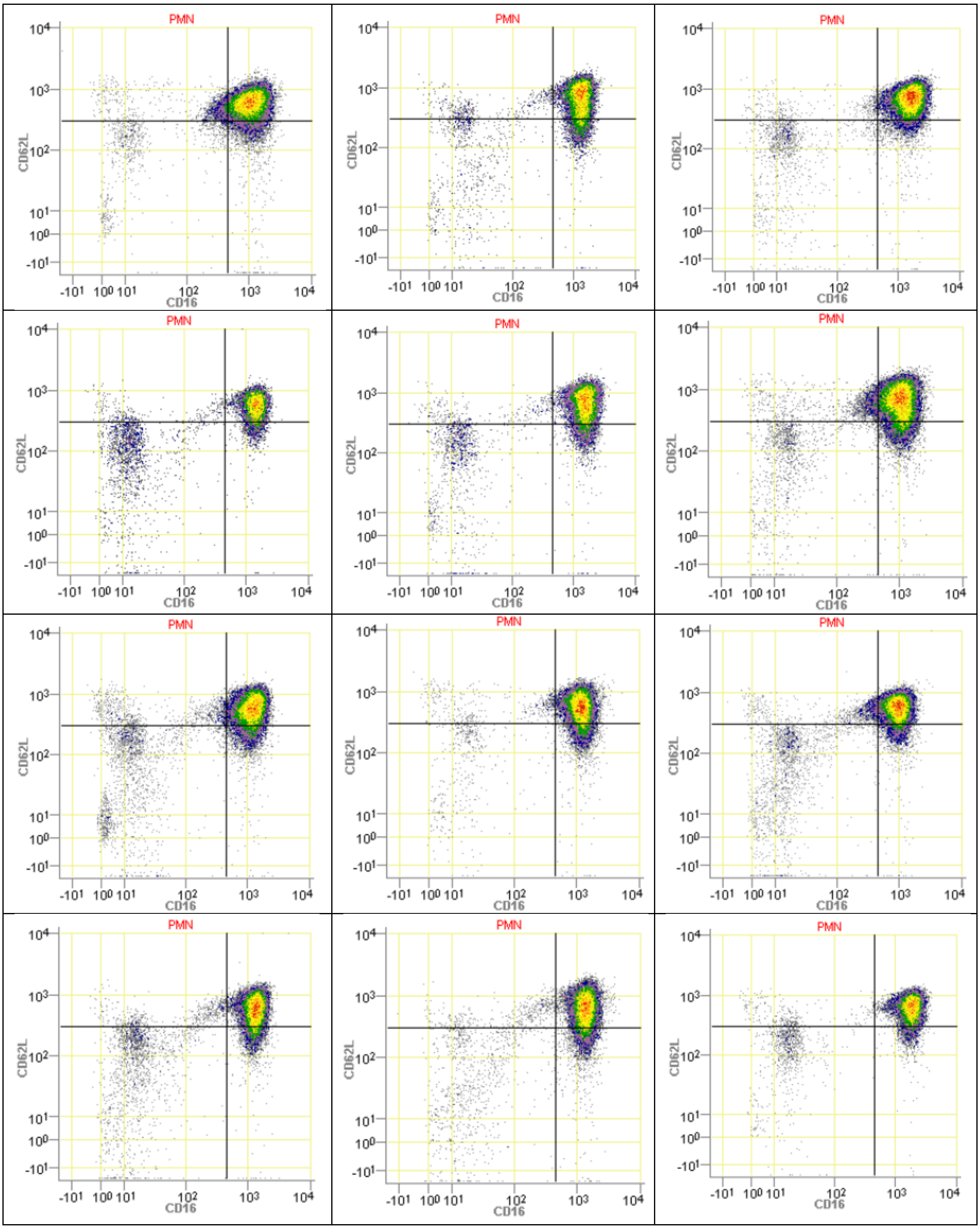
PMN
CD62L
CD16
10^4
10^3
10^2
10^1
10^0
-10^1

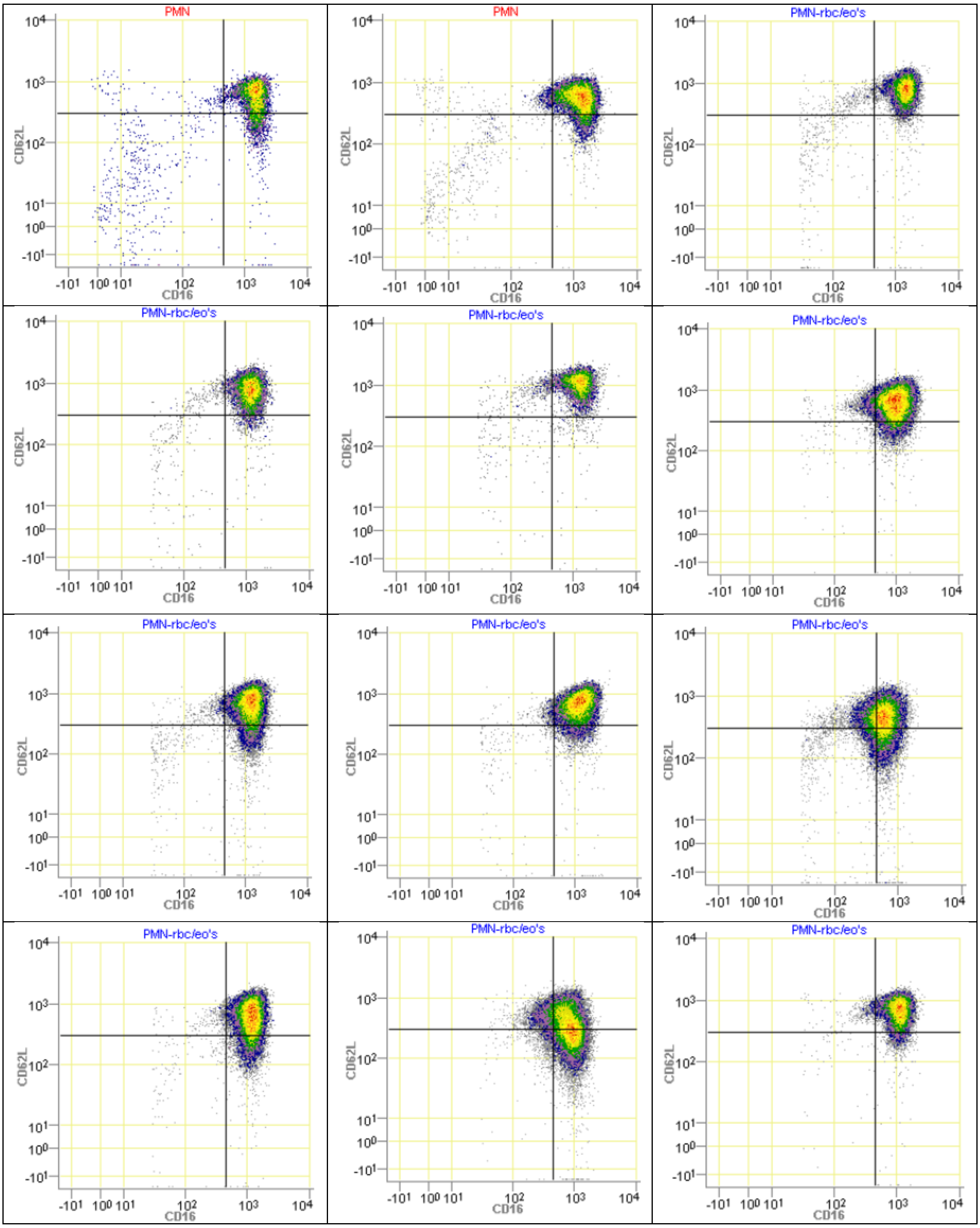
PMN
PMN
PMN-rbc/eo's
PMN-rbc/eo's
PMN-rbc/eo's
PMN-rbc/eo's
PMN-rbc/eo's
PMN-rbc/eo's
PMN-rbc/eo's
PMN-rbc/eo's
PMN-rbc/eo's
PMN-rbc/eo's
CD62L
CD16

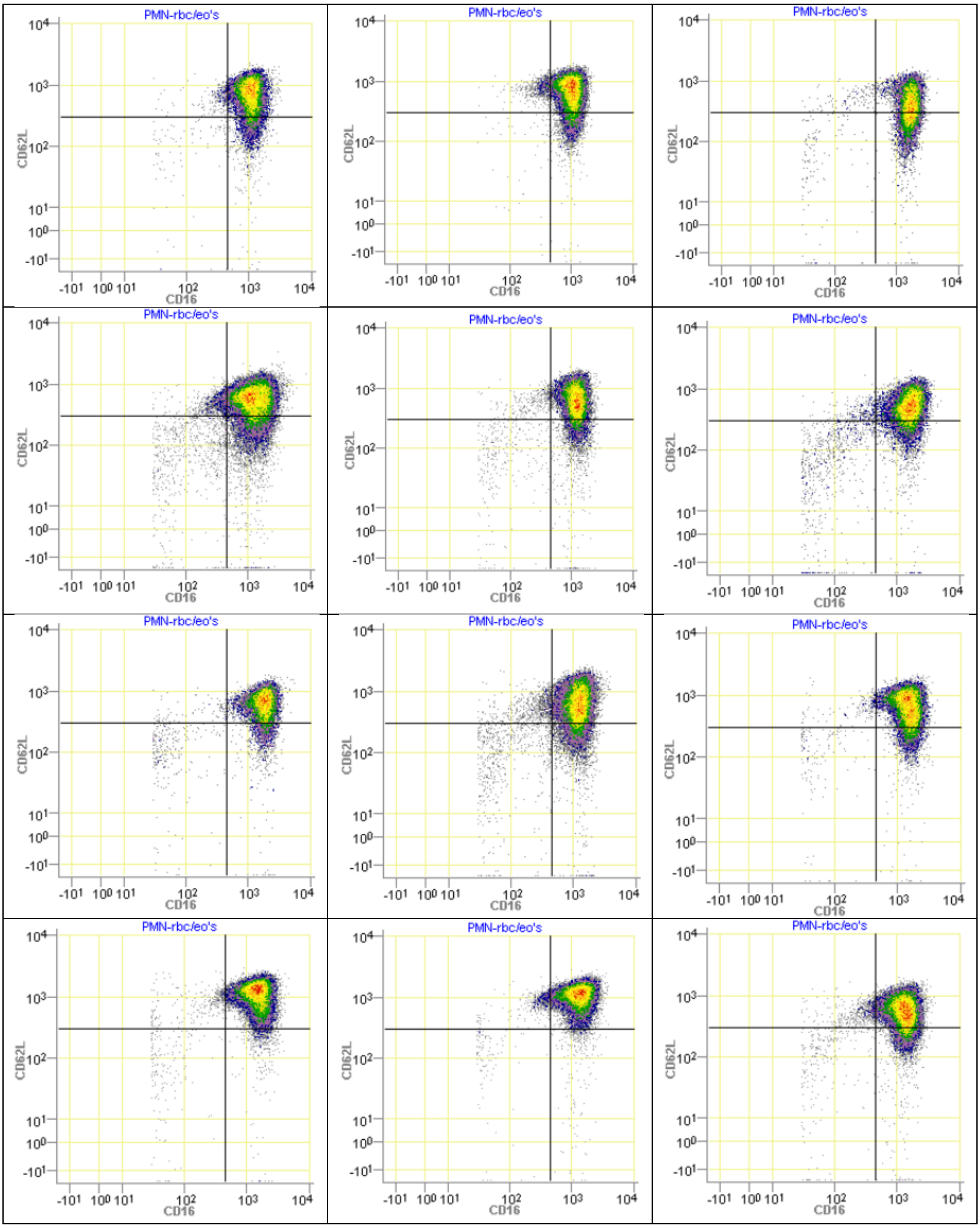
PMN-rbc/eo's
CD62L
CD16
$10^4$
$10^3$
$10^2$
$10^1$
$10^0$
$-10^1$

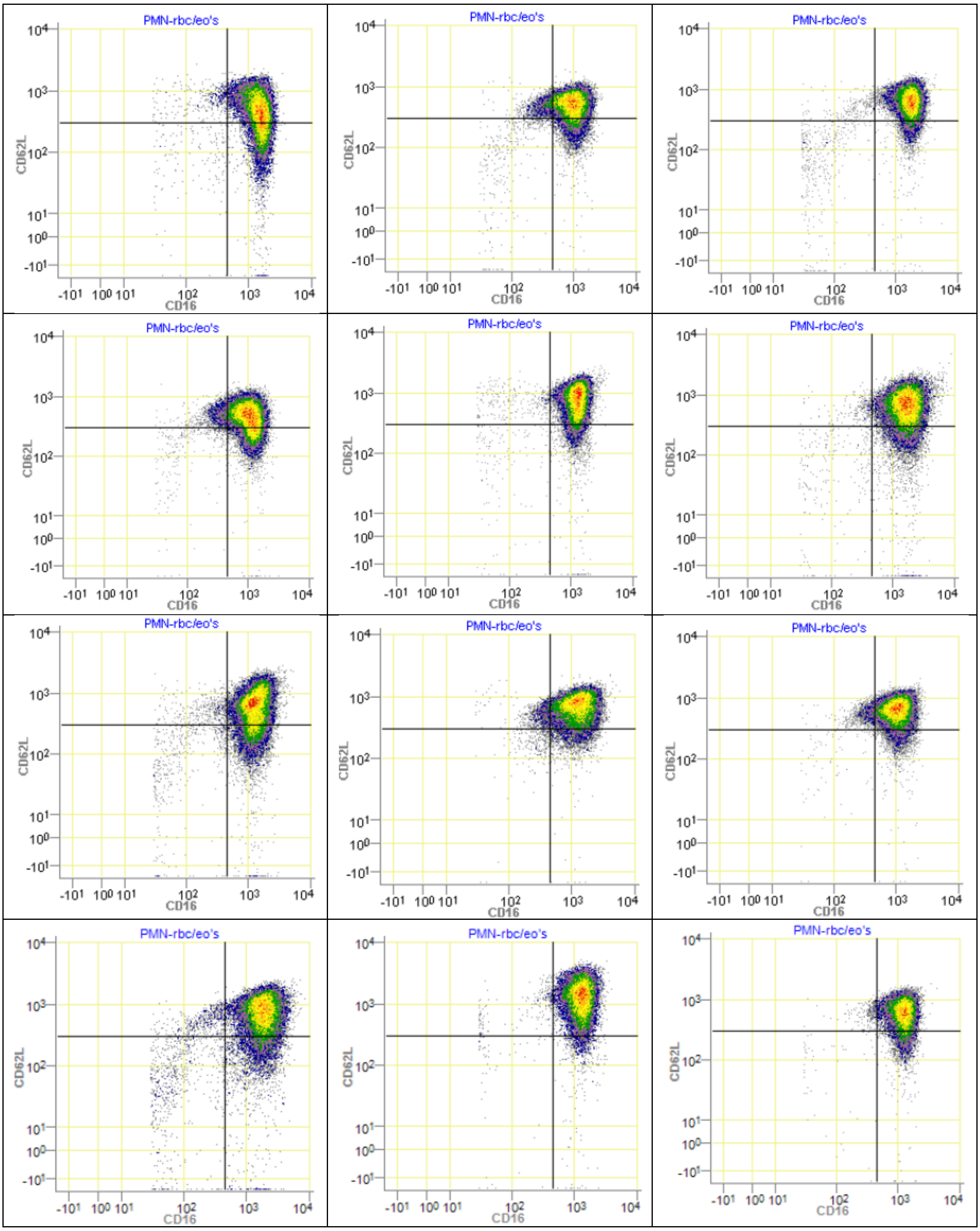
PMN-rbc/eo's
CD62L
CD16
$10^4$
$10^3$
$10^2$
$10^1$
$10^0$
$-10^1$

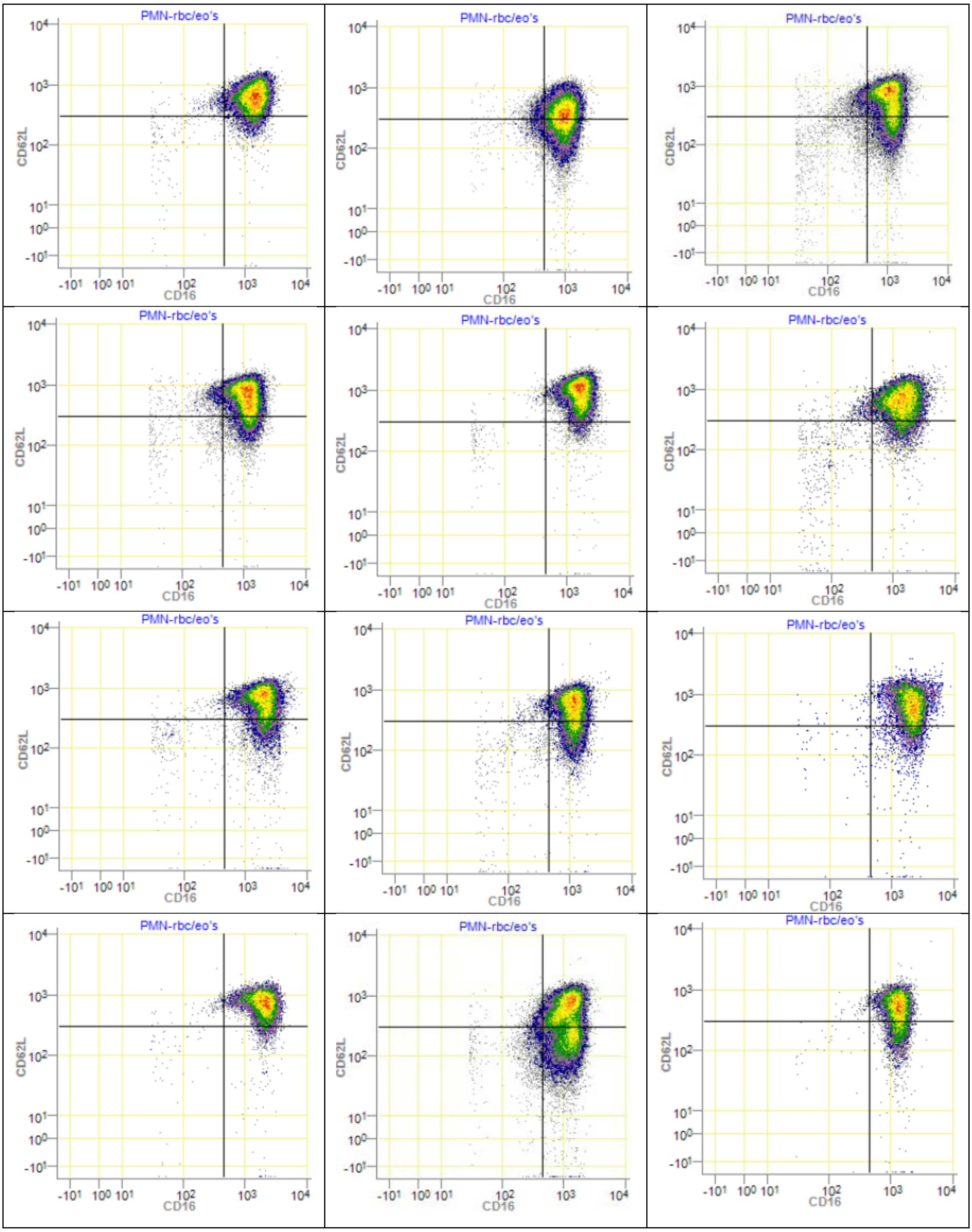
PMN-rbc/eo's
CD62L
CD16
$10^4$
$10^3$
$10^2$
$10^1$
$10^0$
$-10^1$

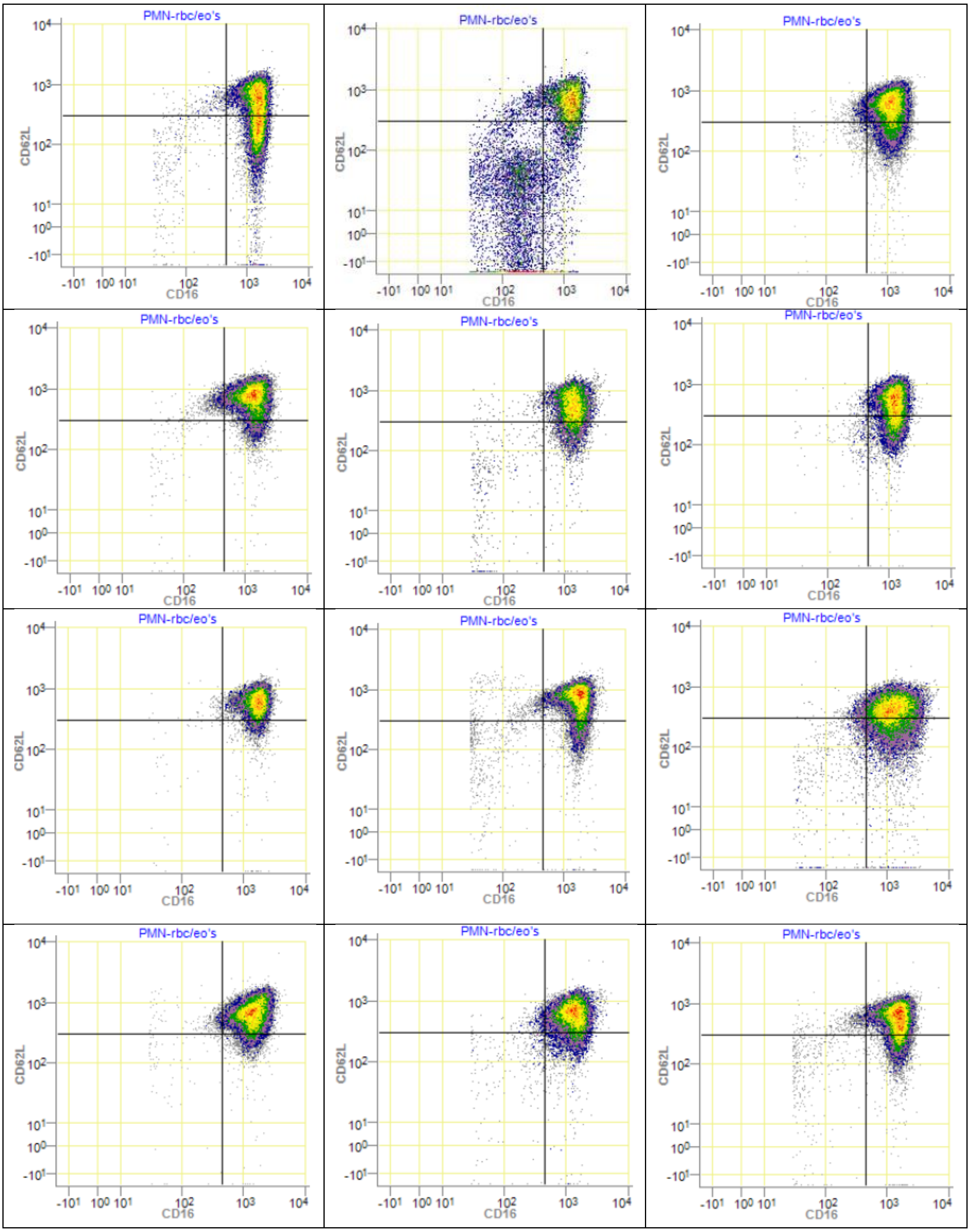
PMN-rbc/eo's
CD62L
CD16
$10^4$
$10^3$
$10^2$
$10^1$
$10^0$
$-10^1$

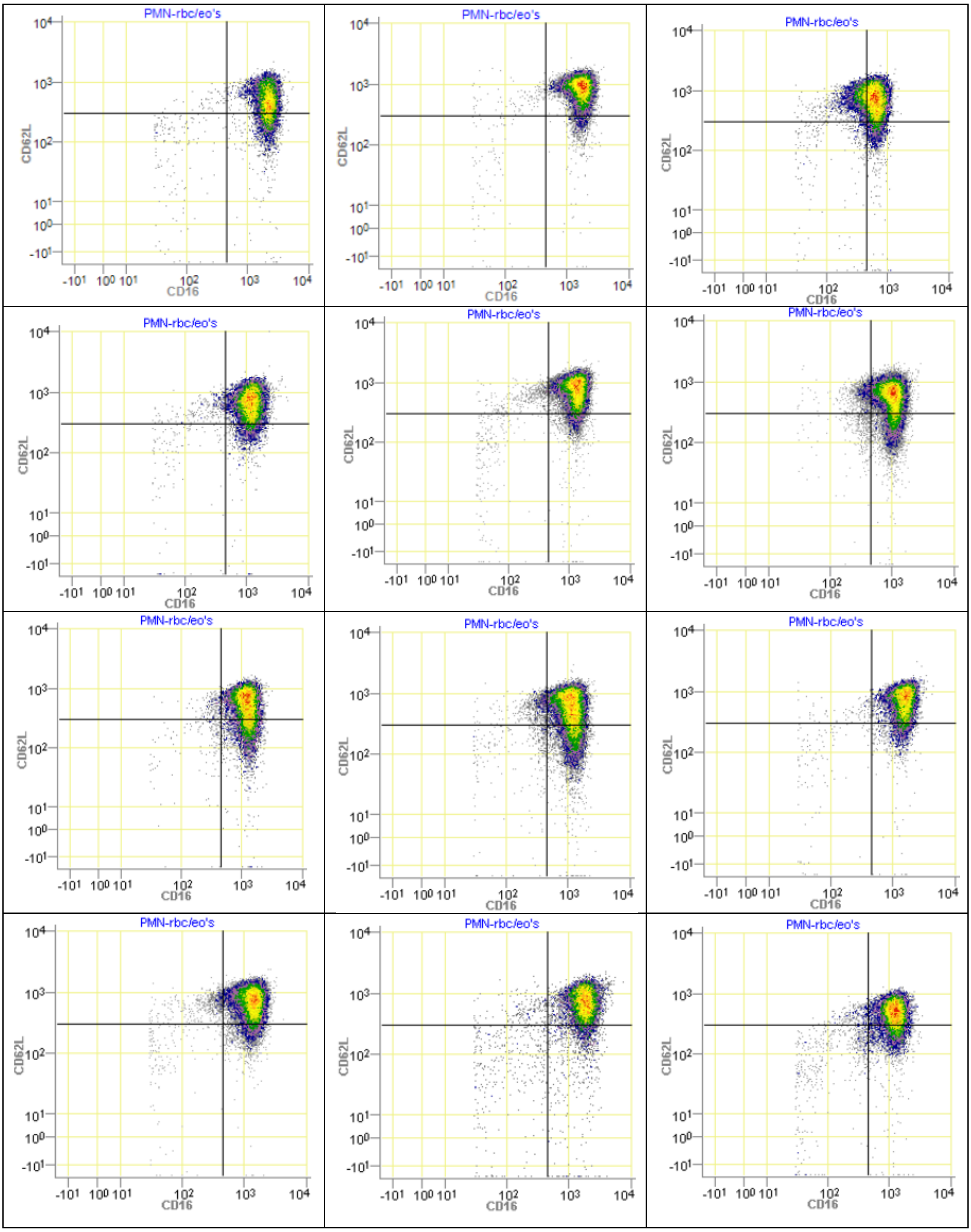
PMN-rbc/eo's
CD62L
CD16

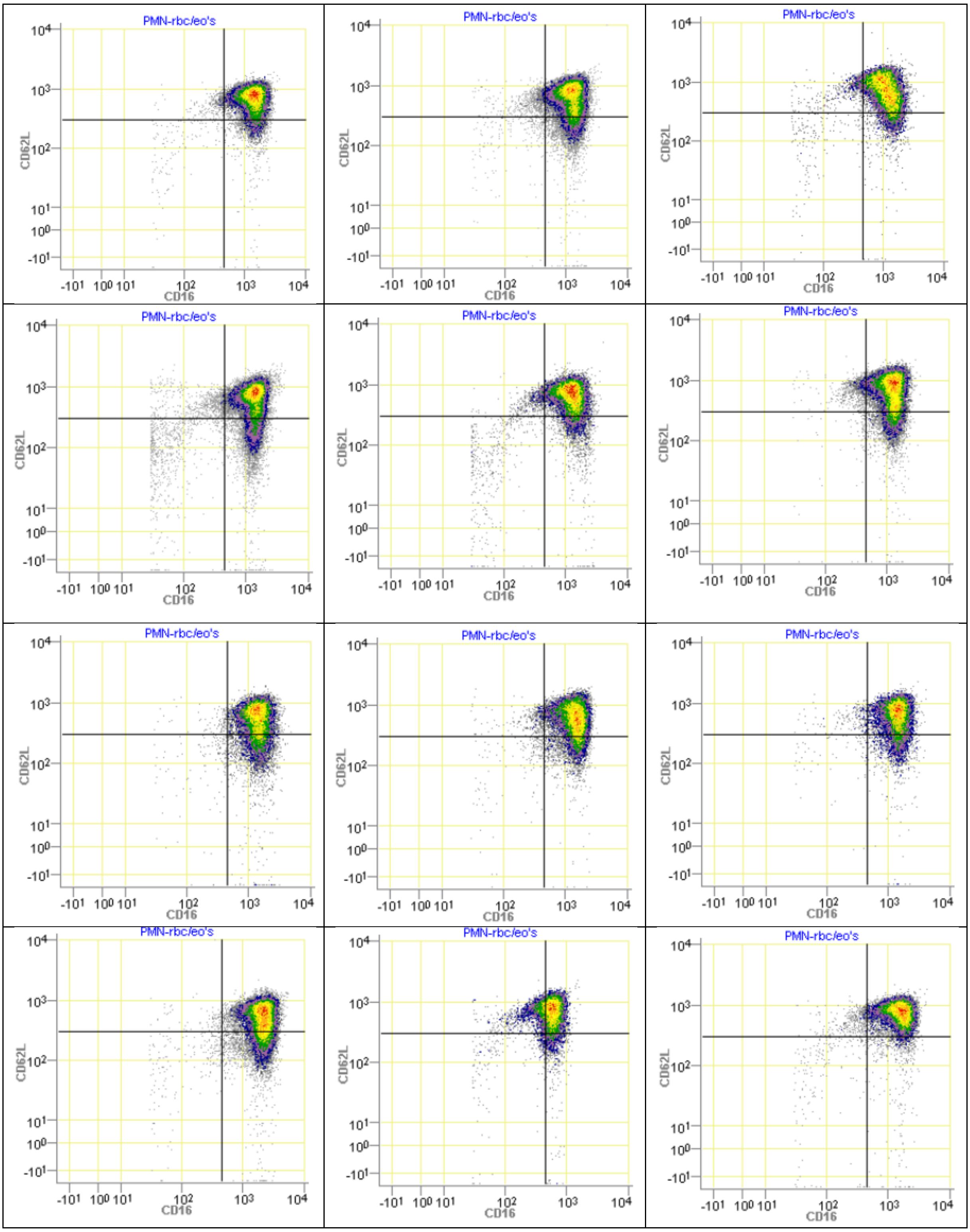
PMN-rbc/eo's
CD62L
CD16
PMN-rbc/eo's
CD62L
CD16
PMN-rbc/eo's
CD62L
CD16
PMN-rbc/eo's
CD62L
CD16
PMN-rbc/eo's
CD62L
CD16
PMN-rbc/eo's
CD62L
CD16
PMN-rbc/eo's
CD62L
CD16
PMN-rbc/eo's
CD62L
CD16
PMN-rbc/eo's
CD62L
CD16
PMN-rbc/eo's
CD62L
CD16
PMN-rbc/eo's
CD62L
CD16
PMN-rbc/eo's
CD62L
CD16

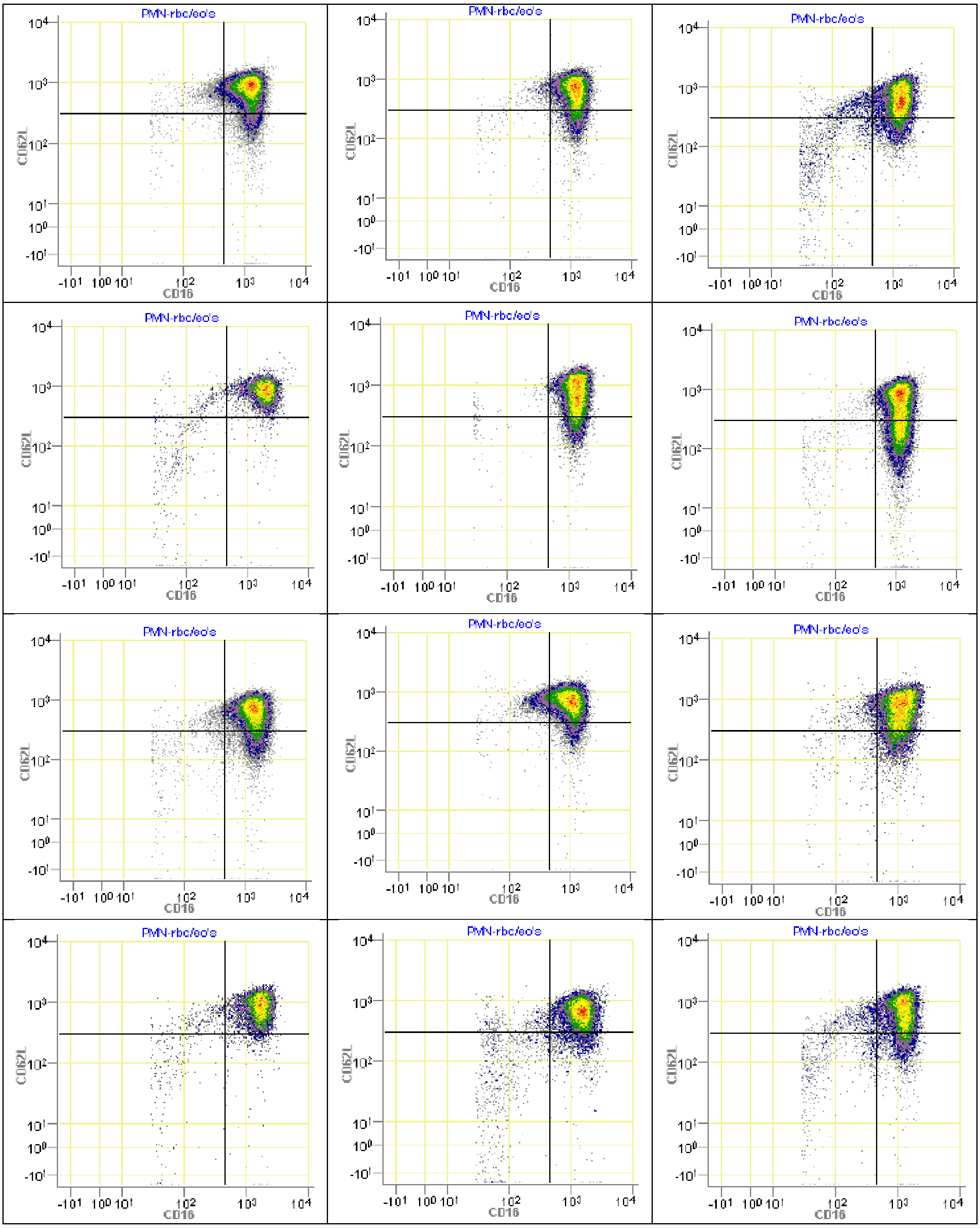
PMN-rbc/eo's
CD62L
CD16
10^4
10^3
10^2
10^1
10^0
-10^1

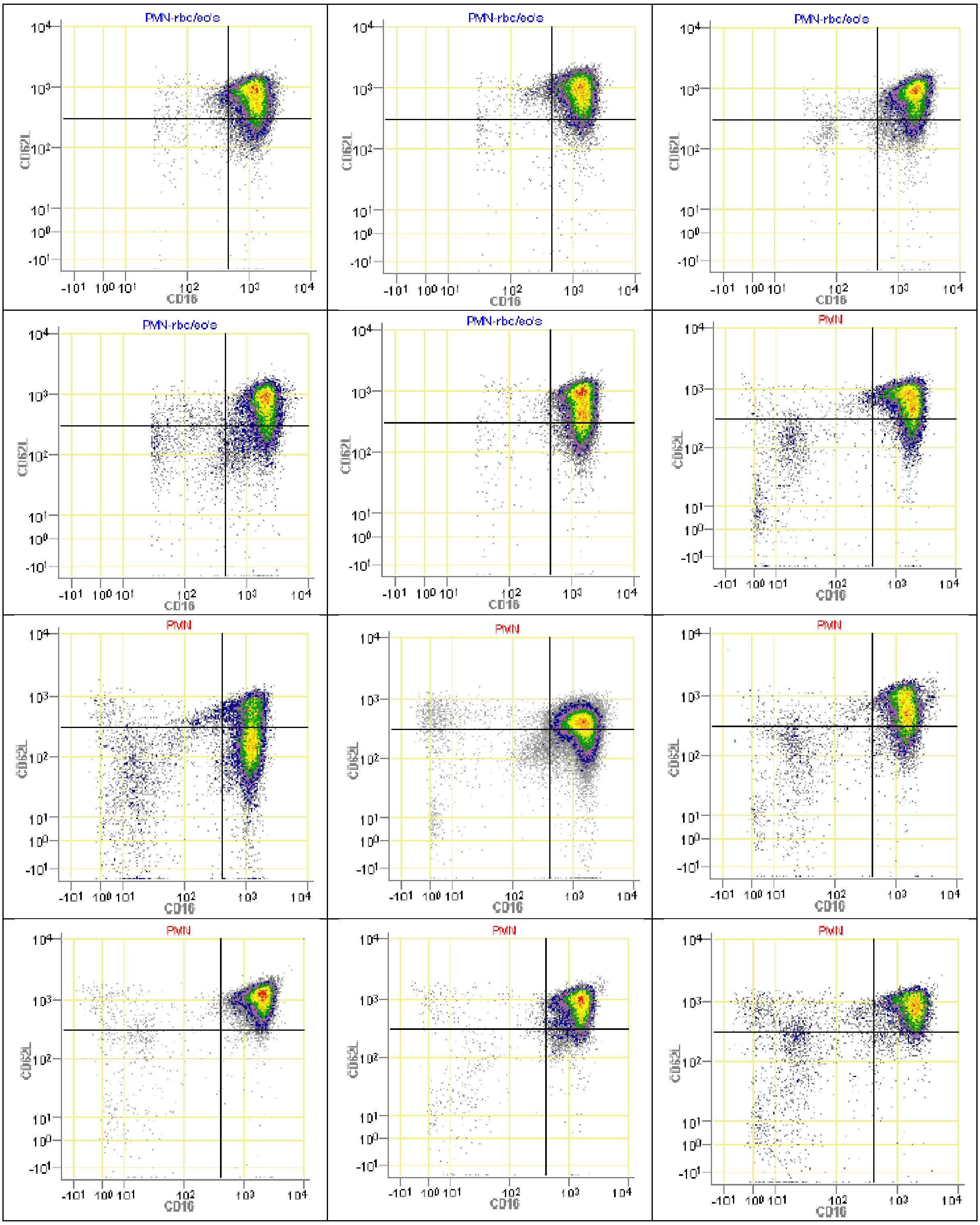
PMN-rbc/eo's
PMN-rbc/eo's
PMN-rbc/eo's
PMN-rbc/eo's
PMN-rbc/eo's
PMN
PMN
PMN
PMN
PMN
PMN
PMN
CD62L
CD16

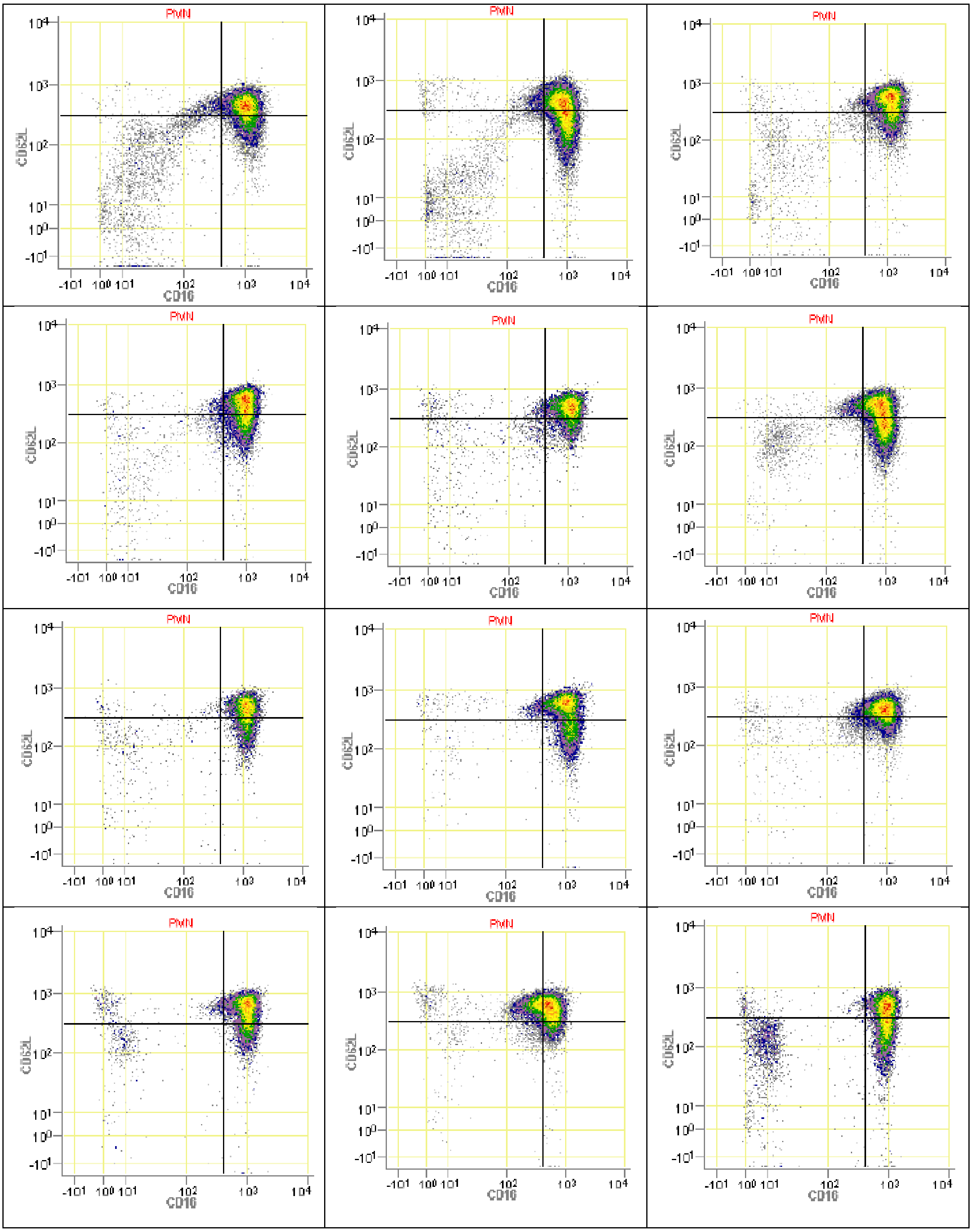
PMN
CD62L
CD16
PMN
CD62L
CD16
PMN
CD62L
CD16
PMN
CD62L
CD16
PMN
CD62L
CD16
PMN
CD62L
CD16
PMN
CD62L
CD16
PMN
CD62L
CD16
PMN
CD62L
CD16
PMN
CD62L
CD16
PMN
CD62L
CD16
PMN
CD62L
CD16

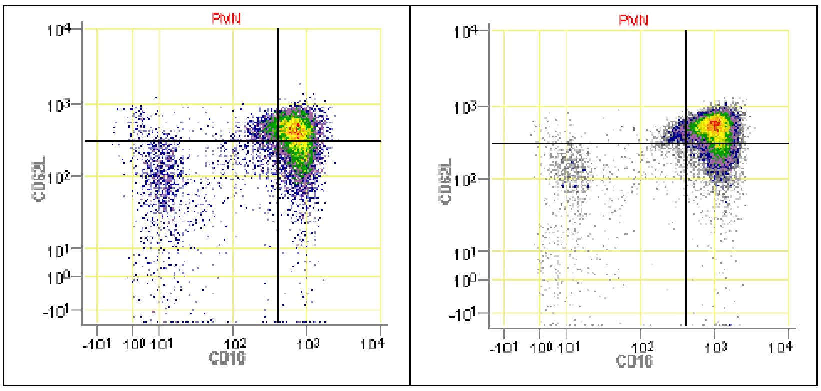
PMN
CD62L
CD16
PMN
CD62L
CD16

# Category 4

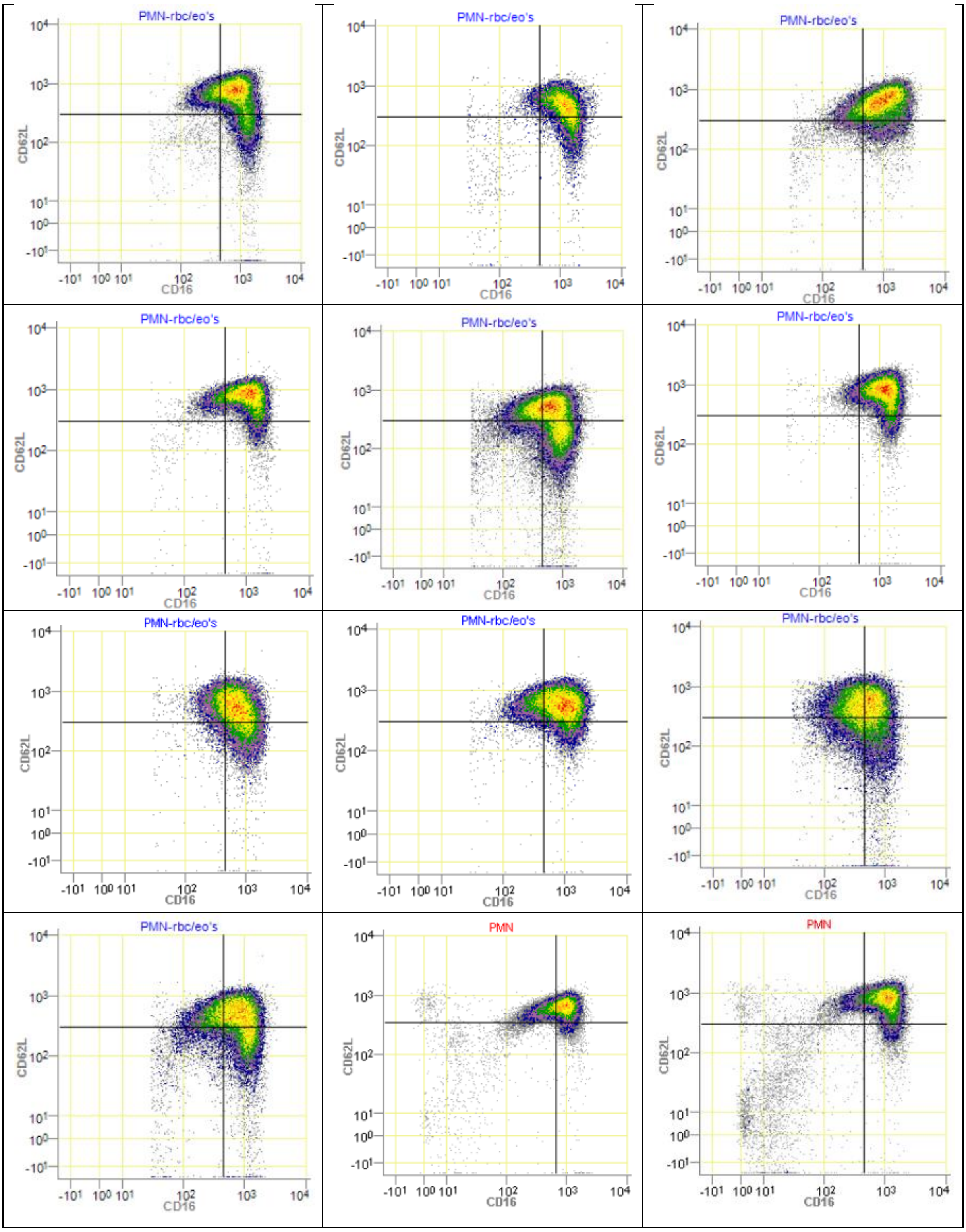

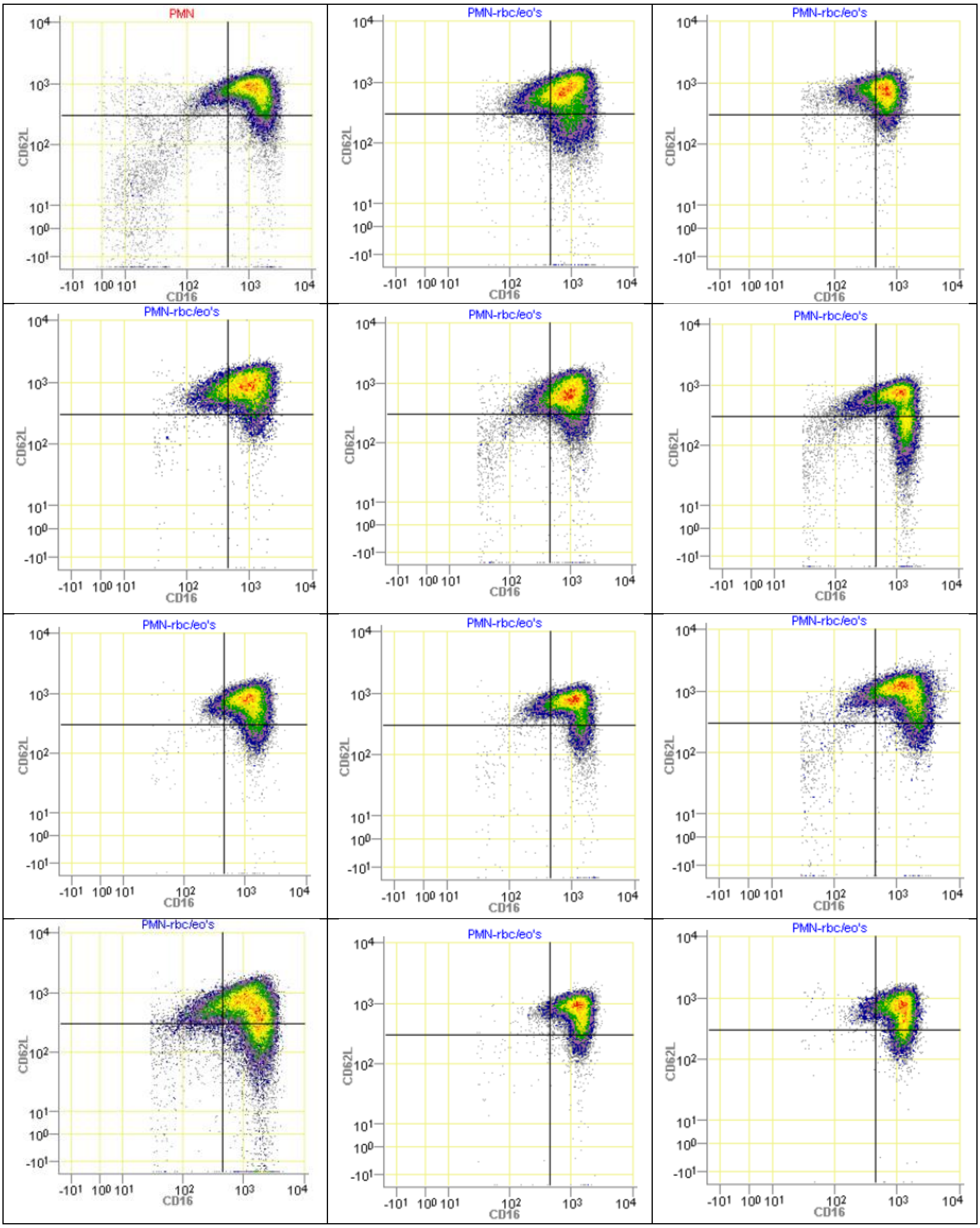
PMN
PMN-rbc/eo's
PMN-rbc/eo's
PMN-rbc/eo's
PMN-rbc/eo's
PMN-rbc/eo's
PMN-rbc/eo's
PMN-rbc/eo's
PMN-rbc/eo's
PMN-rbc/eo's
PMN-rbc/eo's
PMN-rbc/eo's
CD62L
CD16
$10^4$
$10^3$
$10^2$
$10^1$
$10^0$
$-10^1$

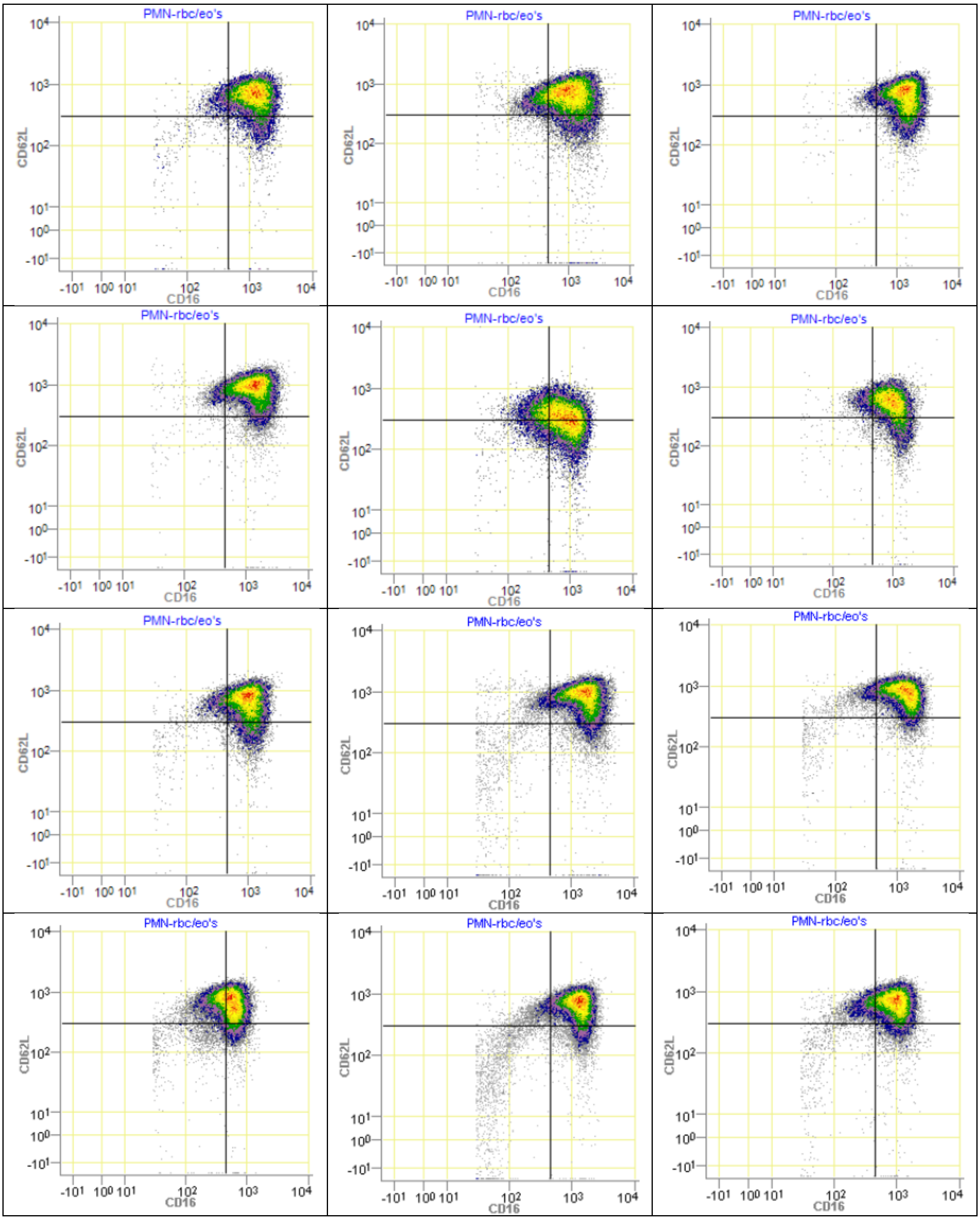
PMN-rbc/eo's
CD62L
CD16
PMN-rbc/eo's
CD62L
CD16
PMN-rbc/eo's
CD62L
CD16
PMN-rbc/eo's
CD62L
CD16
PMN-rbc/eo's
CD62L
CD16
PMN-rbc/eo's
CD62L
CD16
PMN-rbc/eo's
CD62L
CD16
PMN-rbc/eo's
CD62L
CD16
PMN-rbc/eo's
CD62L
CD16
PMN-rbc/eo's
CD62L
CD16
PMN-rbc/eo's
CD62L
CD16
PMN-rbc/eo's
CD62L
CD16

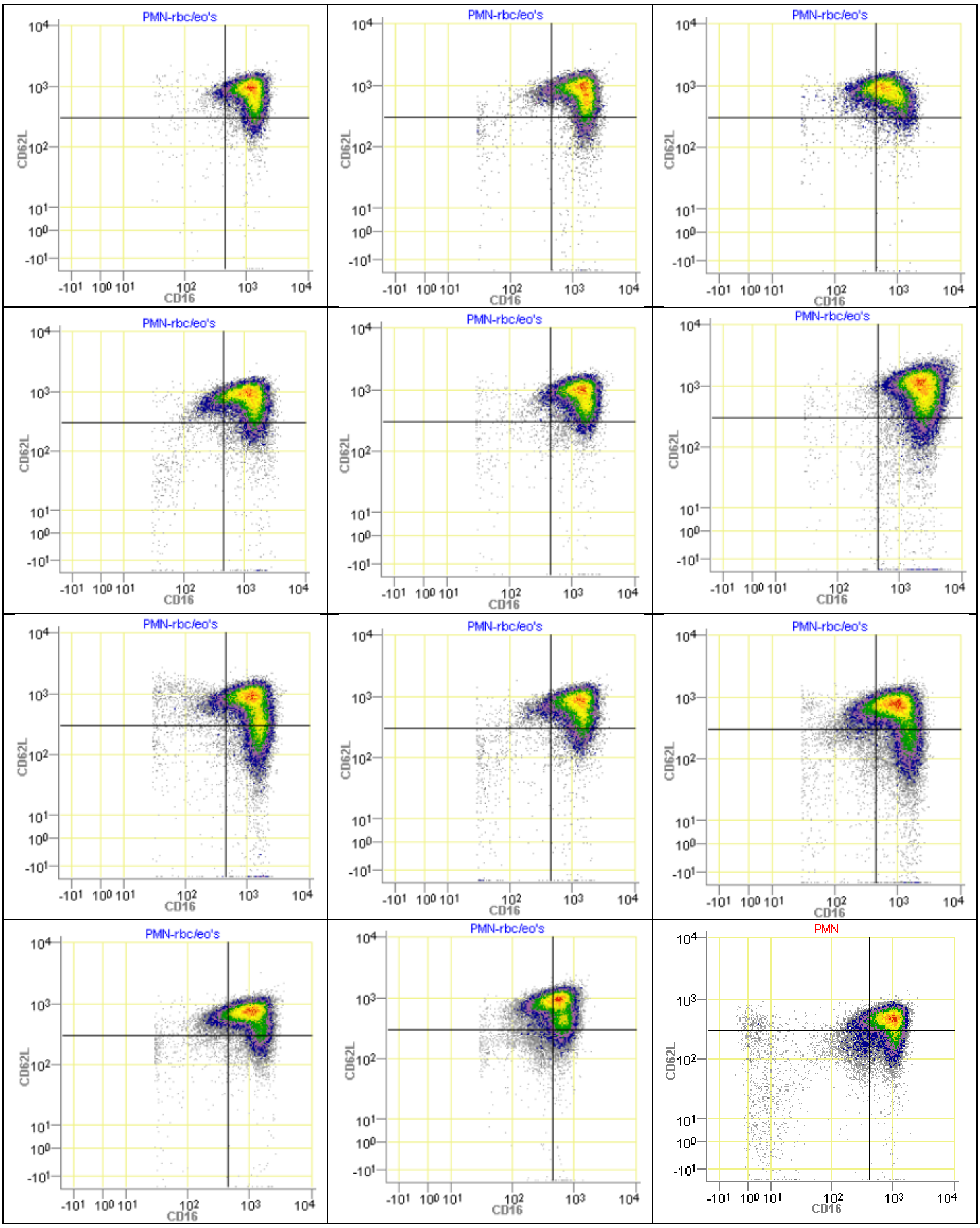
PMN-rbc/eo's
PMN-rbc/eo's
PMN-rbc/eo's
PMN-rbc/eo's
PMN-rbc/eo's
PMN-rbc/eo's
PMN-rbc/eo's
PMN-rbc/eo's
PMN-rbc/eo's
PMN-rbc/eo's
PMN-rbc/eo's
PMN
CD62L
CD16
$10^4$
$10^3$
$10^2$
$10^1$
$10^0$
$-10^1$

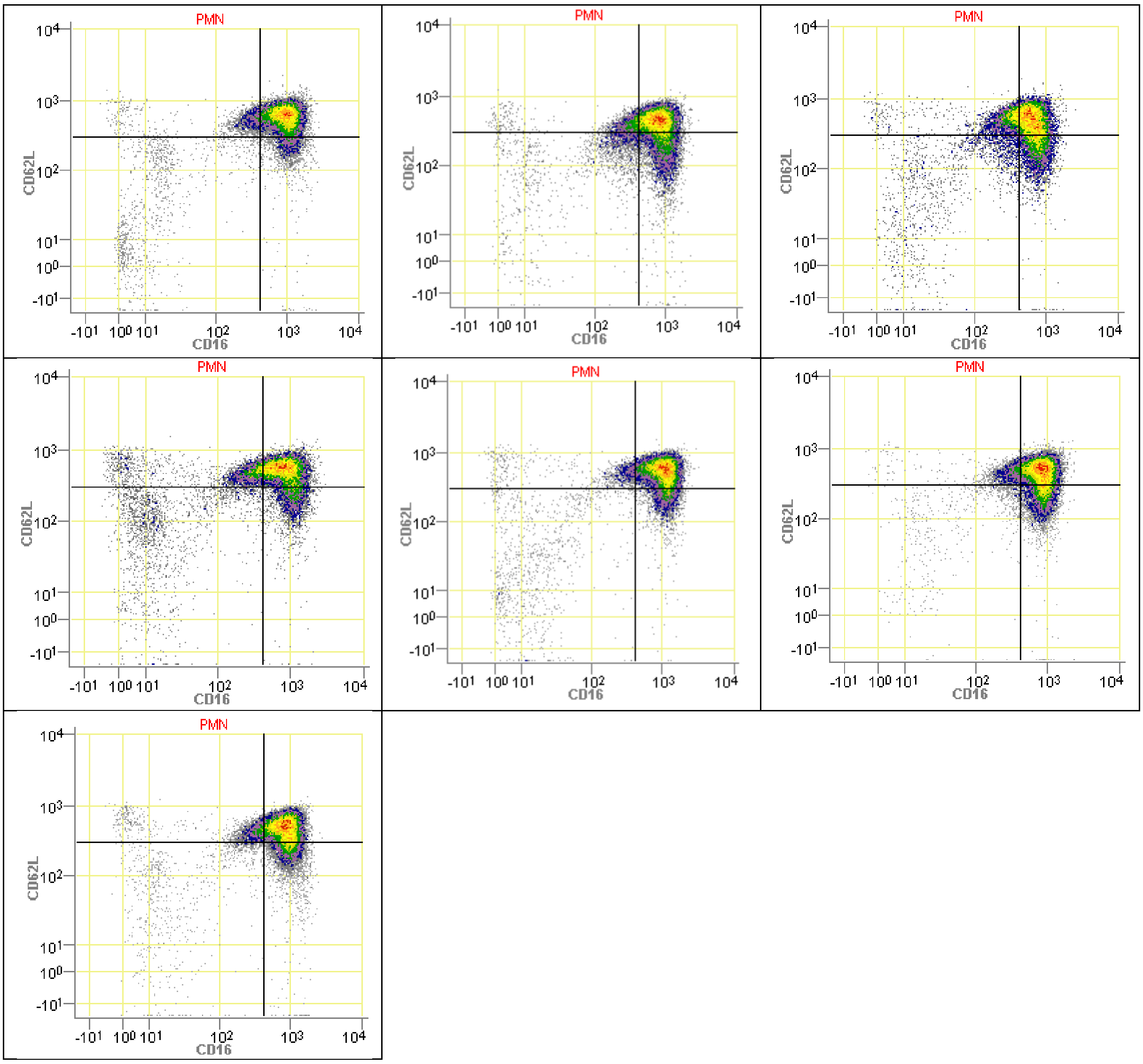
PMN
CD62L
CD16

# Category 5

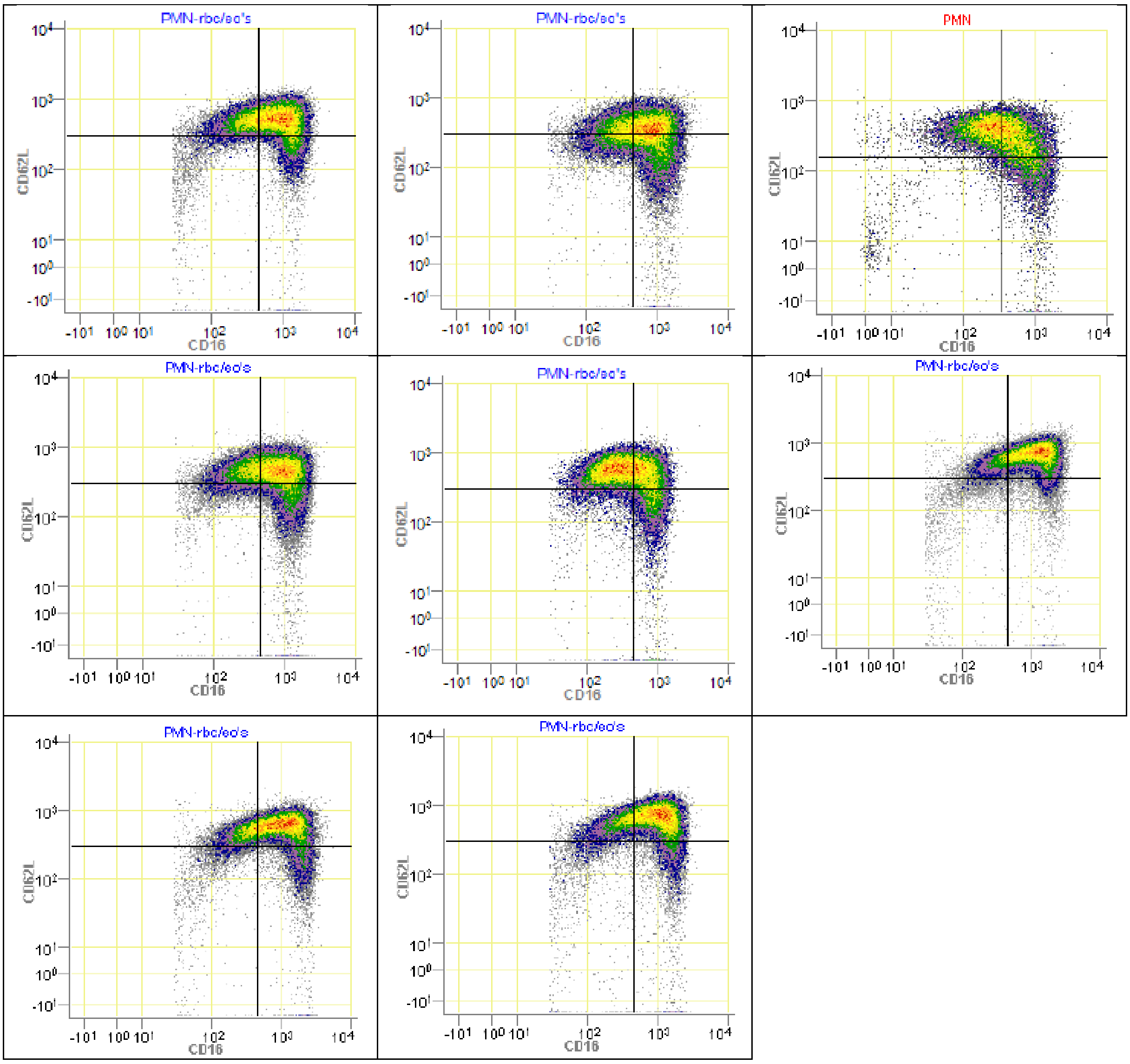
PMN-rbc/eo's
PMN-rbc/eo's
PMN
PMN-rbc/eo's
PMN-rbc/eo's
PMN-rbc/eo's
PMN-rbc/eo's
PMN-rbc/eo's
CD62L
CD16

## Category 6

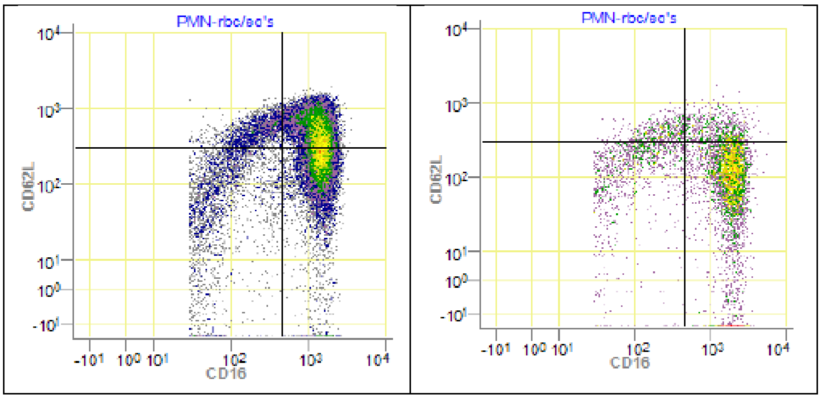

Supplement: Supplementary file 3 — Supplementary file3 Supplementary Material 3 All included individual patient samples (CD16/CD62L dot plots) divided into 7 immunophenotype categories based on the occurrence of subsets of neutrophils in CD16/CD62L dot plots (PDF 6194 KB) [file 68_2022_2134_MOESM3_ESM.pdf]
