# Supplementary material for: Visualization of the inflammatory response to injury by neutrophil phenotype categories: Neutrophil phenotypes after trauma
Source: Eur J Trauma Emerg Surg. 2022 Nov 8;49(2):1023–34. doi: 10.1007/s00068-022-02134-3 (PMC10175373; doi:10.1007/s00068-022-02134-3)
Supplement: Supplementary file 4 — Supplementary file4 Supplementary Material 4 Healthy control samples displaying dot plots of baseline neutrophil CD16/CD62L expression (PDF 679 KB) [file 68_2022_2134_MOESM4_ESM.pdf]

Supplementary Information
Article title: Visualization of the inflammatory response to injury by neutrophil phenotype categories
Journal name: European Journal of Trauma and Emergency Surgery
Author names: E.J. de Fraiture, S.H. Bongers, L. Koenderman, N. Vrisekoop, K.J.P. van Wessem, L.P.H. Leenen, F. Hietbrink.
Corresponding author: F. Hietbrink MD/PhD, Department of Trauma Surgery, University Medical Center Utrecht, The Netherlands. E-mail address: f.hietbrink@umcutrecht.nl

Healthy control

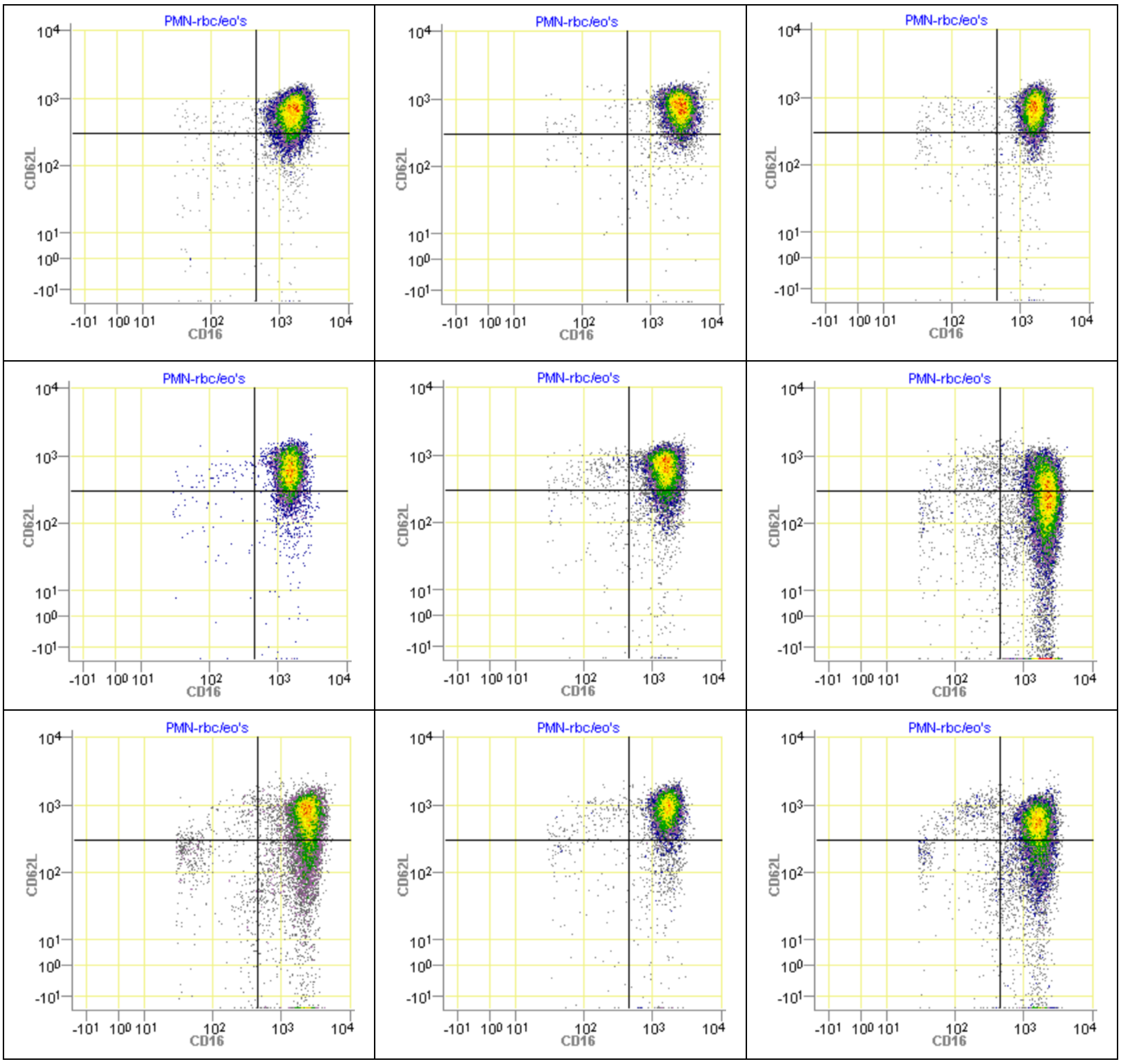

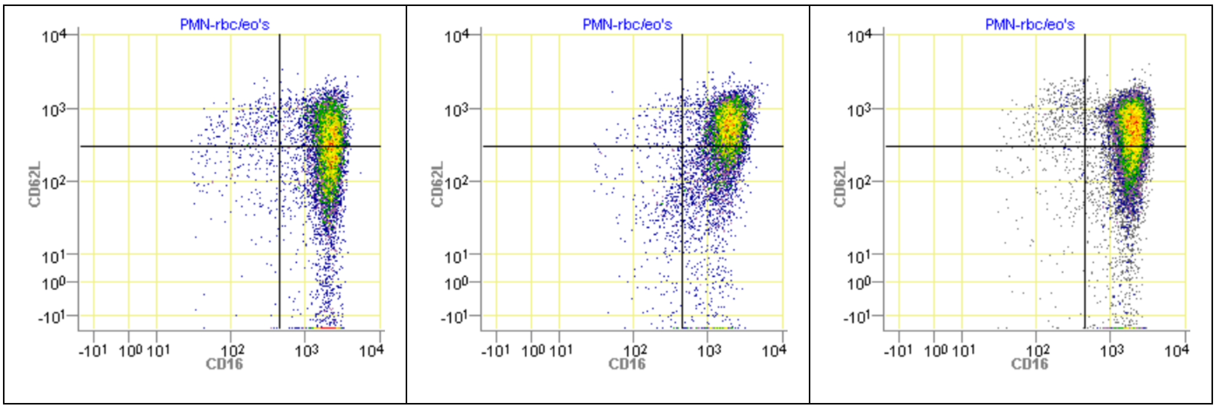
PMN-rbc/eo's
CD62L
CD16
PMN-rbc/eo's
CD62L
CD16
PMN-rbc/eo's
CD62L
CD16
