# Supplementary material for: Visualization of the inflammatory response to injury by neutrophil phenotype categories: Neutrophil phenotypes after trauma
Source: Eur J Trauma Emerg Surg. 2022 Nov 8;49(2):1023–34. doi: 10.1007/s00068-022-02134-3 (PMC10175373; doi:10.1007/s00068-022-02134-3)

Supplementary Information

Article title: Visualization of the inflammatory response to injury by neutrophil phenotype categories

Journal name: European Journal of Trauma and Emergency Surgery

Author names: E.J. de Fraiture, S.H. Bongers, L. Koenderman, N. Vrisekoop, K.J.P. van Wessem, L.P.H. Leenen, F. Hietbrink

Corresponding author: F. Hietbrink MD/PhD, Department of Trauma Surgery, University Medical Center Utrecht, The Netherlands. E-mail address: f.hietbrink@umcutrecht.nl

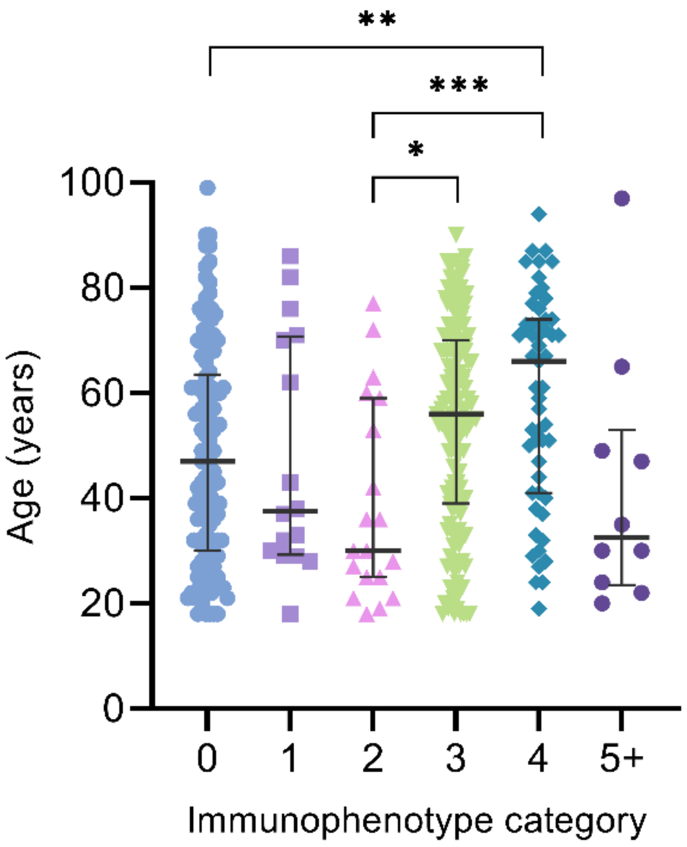

Supplement: Supplementary file 5 — Supplementary file5 Supplementary Material 5 Age (years) of all included patients (group A) in different immunophenotype categories. All flow cytometry measurements executed on admission, during diagnostic work up in the trauma bay (PDF 118 KB) [file 68_2022_2134_MOESM5_ESM.pdf]
