# Supplementary material for: Visualization of the inflammatory response to injury by neutrophil phenotype categories: Neutrophil phenotypes after trauma
Source: Eur J Trauma Emerg Surg. 2022 Nov 8;49(2):1023–34. doi: 10.1007/s00068-022-02134-3 (PMC10175373; doi:10.1007/s00068-022-02134-3)

Supplementary Information

Article title: Visualization of the inflammatory response to injury by neutrophil phenotype categories

Journal name: European Journal of Trauma and Emergency Surgery

Author names: E.J. de Fraiture, S.H. Bongers, L. Koenderman, N. Vrisekoop, K.J.P. van Wessem, L.P.H. Leenen, F. Hietbrink

Corresponding author: F. Hietbrink MD/PhD, Department of Trauma Surgery, University Medical Center Utrecht, The Netherlands. E-mail address: f.hietbrink@umcutrecht.nl

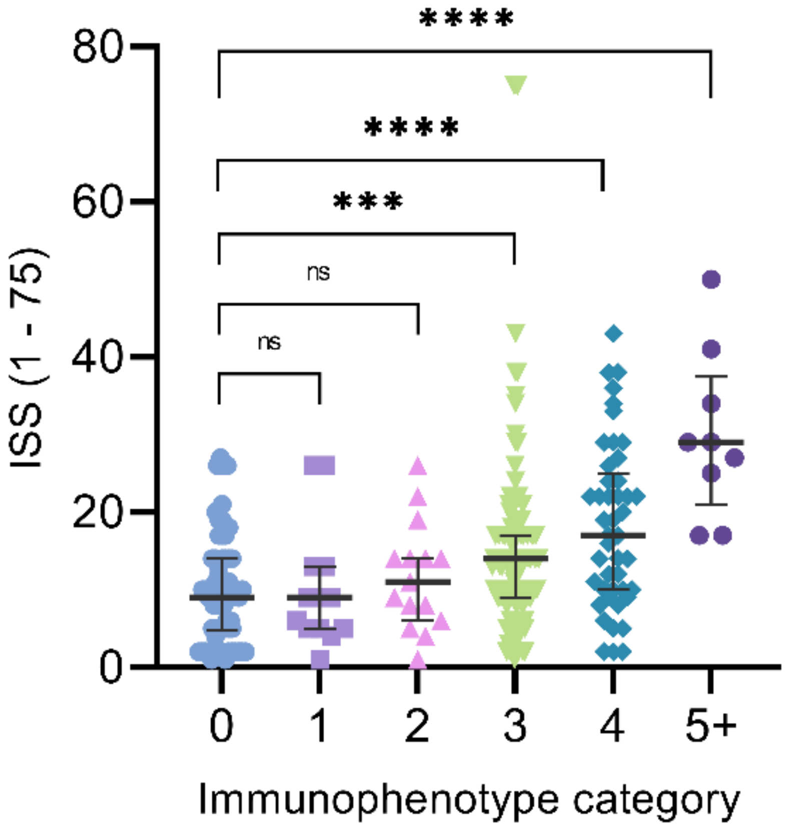

Supplement: Supplementary file 6 — Supplementary file6 Supplementary Material 6 Injury severity score (ISS) of all injured patients (group B) in different immunophenotype categories. All flow cytometry measurements executed on admission, during diagnostic work up in the trauma bay (PDF 109 KB) [file 68_2022_2134_MOESM6_ESM.pdf]
